# Impact of anti‐VEGF therapy versus laser therapy on mortality and treatment outcomes in retinopathy of prematurity: A systematic review and meta‐analysis

**DOI:** 10.1111/aos.17541

**Published:** 2025-06-28

**Authors:** Wei‐De Wang, Ya‐Hsin Yao, Chiao‐Hsin Lan, Shih‐Heng Hung, Ta‐Hsin Tsung, Yann‐Guang Chen, Da‐Wen Lu, Jiann‐Torng Chen, Ke‐Hung Chien, Shu‐I Pao, Wei‐Ting Yen

**Affiliations:** ^1^ Department of Pediatrics Zuoying Armed Forces General Hospital Kaohsiung Taiwan; ^2^ Department of Pediatrics, Tri‐Service General Hospital National Defense Medical Center Taipei Taiwan; ^3^ Department of Ophthalmology, Tri‐Service General Hospital National Defense Medical Center Taipei Taiwan; ^4^ School of Medicine National Defense Medical Center Taipei Taiwan; ^5^ Department of General Medicine, Tri‐Service General Hospital National Defense Medical Center Taipei Taiwan; ^6^ Department of Ophthalmology Show Chwan Memorial Hospital Changhua Taiwan

**Keywords:** laser, retinopathy of prematurity, treatment effect, vascular‐endothelial growth factor

## Abstract

**Purpose:**

Retinopathy of prematurity (ROP) is a major cause of childhood blindness, and selecting the optimal treatment between anti‐vascular endothelial growth factor (anti‐VEGF) and laser therapy is crucial. Understanding their impact on key outcomes, particularly mortality, is essential for informed clinical decision‐making.

**Methods:**

A systematic literature search identified published studies comparing anti‐VEGF and laser therapy for ROP up to December 31, 2024. Primary outcomes included mortality, retinal detachment, surgical interventions, myopia and neurodevelopmental outcomes. The risk of bias was assessed using the Cochrane Risk of Bias Tool and ROBINS‐I. Data were synthesized using a random‐effects model, with risk ratios (RR) and 95% confidence interval (CI). This review is registered in PROSPERO (CRD42024585336).

**Results:**

A total of 12 randomized controlled trials (RCTs) and 58 observational studies, covering 10 516 infants, were included. Anti‐VEGF therapy was associated with a higher mortality risk than laser therapy (RR: 1.68; 95% CI: 1.23–2.30), primarily in observational studies (1.85; 1.32–2.60), while RCTs showed no significant difference (1.02; 0.46–2.26). Anti‐VEGF therapy was linked to lower risks of retinal detachment (0.36; 0.27–0.50), fewer surgical interventions (0.38; 0.22–0.65), and a lower risk of myopia (0.67; 0.54–0.82). No significant differences were found in neurodevelopmental outcomes (1.05; 0.96–1.15).

**Conclusions:**

Anti‐VEGF therapy offers benefits over laser treatment, including reduced retinal detachment, fewer surgeries and lower myopia risk, with no observed increase in mortality or neurodevelopmental impairment. Future large‐scale RCTs are needed to clarify mortality risks while minimising the impact of confounding factors.

## 1 INTRODUCTION

Retinopathy of prematurity (ROP) is a retinal disorder that affects premature infants and is a leading cause of childhood blindness, characterized by abnormal retinal blood vessel development due to dysregulated vascular endothelial growth factor (VEGF) (Alon et al., 1995; Kim et al., 2018). The 2019 Global Burden of Disease Study reported a rising prevalence of ROP‐related vision loss (Wang et al., 2024).

Laser therapy has been a key treatment for ROP, known for its safety and effectiveness (Good et al., 2003, 2004, 2006). It aims to eliminate excessive angiogenic stimulators like VEGF, Tie‐2 and IGF‐1 from the avascular peripheral retina (Imamoglu et al., 2014; Kong, Bhatt, et al., 2015). Identifying VEGF's role in ROP has driven the exploration of anti‐VEGF agents as alternative treatments (Alon et al., 1995). As pegaptanib was first used for retinal diseases (Cunningham Jr et al., 2005; Gragoudas et al., 2004), more anti‐VEGF agents have been developed (Pham et al., 2019). The BEAT‐ROP trial (Mintz‐Hittner et al., 2011) demonstrated a significant benefit of bevacizumab over laser in infants with Zone I disease, while the RAINBOW trial (Stahl et al., 2019) showed a trend towards improved outcomes with ranibizumab compared to laser, though without statistical superiority. These findings support the expanding role of intravitreal anti‐VEGF as a treatment option for ROP.

While both anti‐VEGF and laser therapies are effective for ROP, safety concerns remain. Specifically, studies suggest anti‐VEGF may increase the risk of neurodevelopmental disabilities and pulmonary hypertension compared to laser therapy (Morin et al., 2016; Natarajan et al., 2019; Nitkin et al., 2022). Avery et al. (2016) noted that prolonged anti‐VEGF use for diabetic macular oedema may be linked to higher mortality. Although subsequent meta‐analyses found no significant mortality increase with anti‐VEGF compared to laser in ROP (Sankar et al., 2018; Taher et al., 2022), Natarajan et al. (2019) still reported higher mortality rates associated with anti‐VEGF use.

Recent meta‐analyses suggest that anti‐VEGF may better reduce myopia and delay recurrence in ROP (Ortiz‐Seller et al., 2024; Xu et al., 2023), but evidence on systemic side effects and structural outcomes, such as retinal detachment, remains inconclusive (Chen et al., 2023; Popovic et al., 2021; Sankar et al., 2018; Taher et al., 2022). Hence, we conducted this systematic review and meta‐analysis to comprehensively assess efficacy and safety, offering a balanced view of benefits and risks.

## 2 METHODS

### 2.1 Literature search

This study adhered to the Preferred Reporting Items for Systematic Reviews and Meta‐analyses (Liberati et al., 2009). A literature search was conducted in EMBASE, Ovid‐Medline, Cochrane library and Scopus using appropriate keywords: retinopathy of prematurity, vascular endothelial growth factors, and laser (Search strategies detailed in Table S1). W.D.W completed the final search on December 31, 2024. The review is registered in PROSPERO (CRD42024585336).

### 2.2 Inclusion/exclusion criteria

We included studies according to the following criteria were as follows: (1) randomized controlled trials (RCTs) or observational studies; (2) involved preterm infants with ROP; (3) a comparison between anti‐VEGF and laser therapy; and (4) a report of at least one clinical outcome, such as mortality, retinal detachment, surgical intervention, myopia, developmental delay, etc. The exclusion criteria were as follows: (1) Phase I and II clinical trials, (2) studies including patients with vitreoretinal diseases caused by other than ROP, (3) studies using solely combination therapy with anti‐VEGF and laser, and (4) studies focusing on advanced stages of ROP (Stages 4 and 5). W.D.W and W.T.Y independently reviewed the articles for inclusion using EndNote X9.

### 2.3 Data extraction and quality assessment

W.D.W and W.T.Y independently extracted data and assessed the risk of bias, with S.I.P resolving disagreements. RCTs were evaluated using the Cochrane Risk of Bias Tool 2.0 (Sterne et al., 2019), and observational studies with the ROBINS‐I tool (Sterne et al., 2016). In case of disagreements, S.I.P. made the final decision following Cochrane Handbook guidelines (Higgins et al., 2024).

### 2.4 Data synthesis and analysis

The meta‐analysis was conducted using Review Manager 5.4. The primary outcomes were mortality rate, retinal detachment incidence, need for surgical intervention, myopia incidence and neurodevelopmental consequences (developmental delay or cerebral palsy). The secondary outcomes were unfavourable ocular outcomes, including retinal or macular dragging/traction, retinal holes, macular or retinal fold, macular ectopia, haemorrhage, uveitis, endophthalmitis, cataract or lens opacity, keratitis, corneal erosion and corneal opacity. Outcomes were analysed as dichotomous variables using risk ratios (RR) with 95% confidence intervals (CI), with statistical significance set at *p* < 0.05. Heterogeneity was assessed via Cochran *Q* and *I* ^2^ statistics (*p* < 0.10, *I* ^2^ > 40% indicating significance). Sensitivity analysis tested result robustness, funnel plots assessed publication bias and subgroup analysis compared RCTs and observational studies. A random‐effects model was used due to the diversity in clinical conditions.

## 3 RESULTS

### 3.1 Literature search

**FIGURE 1:**
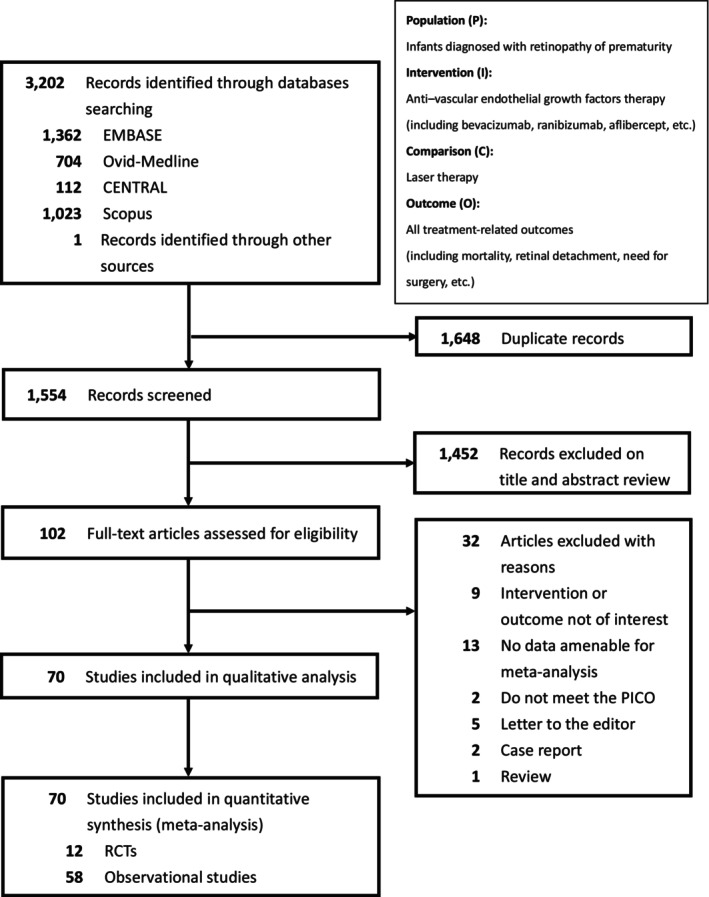
Study selection flowchart.

The literature search and selection process are illustrated in Figure 1. A comprehensive search across multiple databases identified 3202 references, along with one additional reference from other sources. After removing 1648 duplicates and excluding 1452 irrelevant titles/abstracts, 102 full‐text articles were assessed for eligibility, leading to the exclusion of 32 additional studies. The remaining 70 studies met the inclusion criteria and were subjected to qualitative analysis. Ultimately, 70 studies, including 12 RCTs and 58 observational studies, were included in the quantitative meta‐analysis.

### 3.2 Characteristics of the eligible studies

The characteristics of the included studies (12 RCTs and 58 observational studies) are summarized in Table 1. The 70 studies included between 9 and 1577 infants diagnosed with ROP, ranging in gestational age from 23 to 32 weeks. The experimental group consisted of patients who received anti‐VEGF therapy, including aflibercept, bevacizumab, conbercept or ranibizumab. Patients who received laser therapy were assigned to the control group. The follow‐up periods ranged from 1 months to 5 years.

**TABLE 1:** Characteristics of included studies regarding the efficacy and safety outcomes of intravitreal anti‐VEGFs and laser treatment.

	Study	Study design	Country	No. of patient (E/C)	No. of eyes (E/C)	Treatment type (E/C)	Dosage of anti‐VEGF agent	GA (weeks) (E/C)	Follow‐up (months)	Definition of myopia
1	Mintz‐Hittner et al. (2011)	RCT	United States	70/73	140/146	IVB/Laser	IVB (0.625 mg)	24.4/24.4	13.5	NA
2	Harder et al. (2013)	Observational study	Germany	12/13	23/26	IVB/Laser	IVB (0.375 mg or 0.625 mg)	25.2/25.3	12	Moderate myopia ≤−5.00 D High myopia ≤−8.00 D
3	Lepore et al. (2014)	RCT	Italy	NA	13/13	IVB/Laser	IVB (0.5 mg)	25.5^a^	9	NA
4	Geloneck et al. (2014)	Observational study	United States	56/53	110/101	IVB/Laser	IVB (0.625 mg)	24.4/24.3	30	Low myopia −1.00 to >−5.00 D High myopia −5.00 to >−8.00 D Very high myopia ≤−8.00 D
5	Hwang et al. (2015)	Observational study	United States	11/17	22/32	IVB/Laser	IVB (0.625 mg)	24.2/24.8	21.7–34.5	NA
6	Kong, Dinh, et al. (2015)	Observational study	United States	22/20	43/37	IVB/Laser	IVB (0.625 mg)	24.3/24.8	12	NA
7	Gunay et al. (2015)	Observational study	Turkey	25/15	48/30	IVB/Laser	IVB (0.625 mg)	26.4/27.3	19.8	≤−0.25 D
8	Isaac et al. (2015)	Observational study	Canada	13/12	23/22	IVB/Laser	IVB (0.625 mg)	25.2/25.0	12	Myopia ≤−0.25 D High myopia ≤−5.00 D
9	Chan et al. (2016)	Observational study	Hong Kong	4/5	8/10	IVR/Laser	IVR (0.25 mg)	24.2/25.5	20.2–24.7	NA
10	Morin et al. (2016)	Observational study	Canada	27/98	NA	IVB/Laser	NA	24.9/24.7	18	NA
11	Walz et al. (2016)	Observational study	Germany	20/68	38/132	IVB or IVR/Laser	NA	25^a^	NA	NA
12	Gunay, Celik, et al. (2016)	Observational study	Turkey	14/28	27/49	IVB/Laser	IVB (0.625 mg)	26.0/28.0	12	Myopia ≤−0.25 D High myopia ≤−5.00 D
13	Gunay, Sekeroglu, et al. (2016)	Observational study	Turkey	15/21	NA	IVB/Laser	NA	32.2/32.0	17.9–18.6	NA
14	Nicoară et al. (2016)	Observational study	Romania	17/6	34/12	IVB/Laser	IVB (0.625 mg)	28.2/29.8	15–20	NA
15	O'Keeffe et al. (2016)	RCT	Ireland	NA	15/15	IVB/Laser	IVB (1.25 mg)	25^a^	60	NA
16	Karkhaneh et al. (2016)	RCT	Iran	43/36	86/72	IVB/Laser	IVB (0.625 mg)	28.3/28.5	22.5	NA
17	Kabataş et al. (2017)	Observational study	Turkey	18/36	36/72	IVB and IVR/Laser	IVB (0.625 mg) /IVR (0.25 mg)	26.0/27.7	18	NA
18	Mueller et al. (2017)	Observational study	Germany	37/17	74/34	IVB/Laser	IVB (0.625 mg)	25^a^	12–15	NA
19	Gunay et al. (2017)	Observational study	Turkey	77/57	151/113	IVB and IVR/Laser	IVB (0.625 mg)/IVR (0.25 mg)	27.4/28.2	18.9–20.6	Myopia ≤−0.25 D High myopia ≤−5.00 D
20	Lolas et al. (2017)	Observational study	Chile	23/49	46/98	IVB/Laser	IVB (0.625 mg)	25.2/26.2	10	NA
21	Vujanovic et al. (2017)	Observational study	Serbia	21/45	42/90	IVB/Laser	IVB (0.625 mg)	29.0/30.0	9	Myopia ≤−1.00 D High myopia ≤−3.00 D
22	Zhang et al. (2017)	RCT	China	25/25	50/50	IVR/Laser	IVR (0.3 mg)	28.9/28.2	6	NA
23	Lepore et al. (2018)	RCT	Italy	NA	21/21	IVB/Laser	IVB (0.5 mg)	25.5^a^	48	NA
24	Morrison et al. (2018)	Observational study	United States Canada	14/492	26/963	IVB/Laser	NA	25.0/25.0	4.5	NA
25	Adams et al. (2018)	Observational study	United Kingdom	14/153	22/285	IVB and IVR/Laser	NA	25.1/24.1	12	Myopia ≤−0.25 D High myopia ≤−5.00 D
26	Kang et al. (2018)	Observational study	Korea	NA	153/161	IVR/Laser	IVR (0.25 mg)	27.3/28.8	36.3	NA
27	Walz et al. (2018)	Observational study	Germany	40/97	78/199	IVB and IVR/Laser	NA	25^a^	NA	NA
28	Kennedy et al. (2018)	Observational study	United States	7/9	14/18	IVB/Laser	IVB (0.625 mg)	25.0/24.4	18–28	NA
29	Blair et al. (2018)	Observational study	United States	12/7	22/14	IVB/Laser	IVB (0.5–0.625 mg)	24.5/24.7	28.5–60.7	NA
30	Arfat (2018)	Observational study	Ireland	35/35	NA	IVB/Laser	NA	24.4/24.7	6	NA
31	Roohipoor et al. (2018)	Observational study	Iran	NA	724/262	IVB/Laser	IVB (0.625 mg)	NA	24.1–27.2	NA
32	Leng et al. (2018)	Observational study	China	12/49	NA	IVR/Laser	IVR (0.25 mg)	28.7/31.3	13.5	NA
33	Stahl et al. (2019)	RCT	International	149/69	298/138	IVR/Laser	IVR (0.1 or 0.2 mg)	25.5/26.0	6	NA
34	Natarajan et al. (2019)	Observational study	United States	181/224	NA	IVB/Laser	NA	29.0/28.0	18–26	NA
35	Barry et al. (2019)	Observational study	United States	18/97	36/186	IVB/Laser	IVB (0.375–0.625 mg)	25.1/25.3	5	NA
36	Kang et al. (2019)	Observational study	Korea	12/15	22/30	IVB and IVR/Laser	IVB (0.625 mg)/IVR (0.2 mg)	27.4/34.0	48	Low myopia −1.00 to >−5.00 D High myopia −5.00 to >−8.00 D Very high myopia ≤−8.00 D
37	Raghuram et al. (2019)	Observational study	Canada	34/30	60/51	IVB/Laser	IVB (0.625 mg)	24.4/25.1	18–24	Myopia ≤−0.25 D High myopia ≤−5.00 D
38	Lyu et al. (2019)	Observational study	China	9/5	17/10	IVR/Laser	IVR (0.25 mg)	29.0/28.8	11–15.2	NA
39	Shah et al. (2019)	Observational study	India	115/84	230/168	Anti‐VEGF/Laser	NA	30.1/31.5	NA	NA
40	Roohipoor et al. (2019)	RCT	Iran	77/39	154/78	IVB/Laser	IVB (0.625 mg)	28.7/28.3	22.5	NA
41	Rodriguez et al. (2019)	Observational study	United States	46/40	NA	IVB/Laser	NA	24.7/25.4	24	NA
42	Demir et al. (2019)	Observational study	Turkey	NA	57/64	IVB/Laser	IVB (0.625 mg)	28.4^a^	11	NA
43	Ekinci and Çelik (2020)	Observational study	Turkey	12/15	24/27	IVA/Laser	IVA (1 mg)	27.3/28.7	11.3–11.7	NA
44	Barry et al. (2020)	Observational study	United States	19/119	NA	IVB/Laser	NA	25.1/25.4	0.9	NA
45	Ling et al. (2020)	Observational study	Taiwan	143/33	279/61	IVB and IVR/Laser	IVB (0.625 mg)/IVR (0.25 mg)	26.3/26.5	33.2–60.7	NA
46	Zayek et al. (2020)	Observational study	United States	61/85	NA	IVB/Laser	IVB (0.625 mg)	23.0/24.0	24	NA
47	Zhang et al. (2020)	Observational study	United States	63/235	NA	Anti‐VEGF/Laser	NA	NA	24	NA
48	Chmielarz‐Czarnocińska et al. (2021)	Observational study	Poland	7/2	13/3	IVR/Laser	IVR (0.25 mg)	24.5/25.0	36	≤ −0.50 D
49	Kumari et al. (2021)	Observational study	India	13/10	26/19	Anti‐VEGF/Laser	NA	29.7/31.6	3	NA
50	Barry et al. (2021)	Observational study	United States	NA	164/1003	Anti‐VEGF/Laser	NA	24.6/24.9	15	NA
51	Simmons et al. (2021)	Observational study	United States	22/26	44/52	IVB/Laser	IVB (0.625 mg)	24.5/24.7	42	Myopia ≤−1.00 D High myopia ≤−5.00 D
52	Murakami et al. (2021)	Observational study	Japan	12/14	24/28	IVB/Laser	IVB (0.625 mg)	26.8/25.7	60	≤−0.50 D
53	Marlow et al. (2021)	RCT	International	126/54	254/108	IVR/Laser	IVR (0.1 or 0.2 mg)	NA	24	Myopia ≤−0.25 D High myopia ≤−5.00 D
54	Demir et al. (2021)	Observational study	Turkey	19/12	38/24	IVB/Laser	IVB (0.625 mg)	29.4/29.0	18	Myopia ≤−0.25 D High myopia ≤−5.00 D
55	Mori et al. (2021)	Observational study	Japan	13/53	14/80	IVB/Laser	IVB (0.625 mg)	24.7^a^	12	NA
56	Elabbasy et al. (2022)	Observational study	Saudi Arabia	21/14	42/27	IVR//Laser	IVR (0.25 mg)	25.4/26.2	12	Severe myopia ≤−6.00 D
57	Stahl et al. (2022)	RCT	International	75/38	146/72	IVA/Laser	IVA (0.4 mg)	26.4/26.0	6	NA
58	Gundlach et al. (2022)	Observational study	United States	NA	15/59	Anti‐VEGF/Laser	NA	28.2^a^	72	Severe myopia ≤−5.00 D
59	Nitkin et al. (2022)	Observational study	United States	888/689	NA	Anti‐VEGF/Laser	NA	NA	3.8	NA
60	Linghu et al. (2022)	Observational study	China	642/220	1224/403	IVB and IVR and IVC/Laser	IVB (0.625 mg)/IVR (0.25 mg)/IVC (0.25 mg)	28.9/29.9	6	NA
61	Chou et al. (2022)	Observational study	Taiwan	23/5	NA	IVB/Laser	IVB (0.625 mg)	25.9/26.0	12–24	NA
62	Ahn et al. (2022)	Observational study	Korean	47/189	NA	IVB/Laser	NA	26.7^a^	20	NA
63	Celik et al. (2022)	Observational study	Turkey	12/32	NA	IVB/Laser	NA	27.0/26.0	18.5–32.5	NA
64	Yenice et al. (2023)	Observational study	Turkey	15/30	27/59	IVB/Laser	IVB (0.625 mg)	26.1/26.8	12	NA
65	Stahl et al. (2024)	RCT	International	66/34	128/64	IVA/Laser	IVA (0.4 mg)	26.0^a^	24	High myopia ≤−5.00 D Very high myopia ≤−8.00 D
66	Pfeil et al. (2024)	Observational study	Germany	142/202	276/396	IVB and IVR/Laser	NA	24.6/25.1	NA	NA
67	Winter et al. (2024)	Observational study	Germany	NA	82/43	IVB and IVR/Laser	NA	25.0^a^	NA	NA
68	Tomioka et al. (2024)	Observational study	Japan	6/7	11/13	Anti‐VEGF/Laser	NA	24.6/24.6	62.5	Myopia ≤−0.50 D High myopia ≤−6.00 D
69	Marlow et al. (2024)	RCT	International	126/54	NA	IVR/Laser	IVR (0.1 or 0.2 mg)	25.5/26.0	60	High myopia ≤−5.00 D
70	Wardati et al. (2024)	Observational study	Malaysia	21/25	42/50	IVR/Laser	IVR (0.25 mg)	28.0/27.0	12	NA

Abbreviations: anti‐VEGF, anti‐vascular endothelial growth factor; C, control group; D, diopters; E, experimental group; GA, gestational age; IVA, intravitreal aflibercept; IVB, intravitreal bevacizumab; IVC, intravitreal conbercept; IVR, intravitreal ranibizumab; L, laser; NA, not available; RCT, randomized controlled trial.

^a^ Mean of experimental group and control group.

### 3.3 Quality of the included studies

The 12 included RCTs were evaluated using the Cochrane risk‐of‐bias tool 2.0 (Sterne et al., 2019), with most showing a low risk of bias across five core domains, except for one study (O'Keeffe et al., 2016) classified as high risk (Table S2). The 61 observational studies, including retrospective and prospective cohorts, were assessed with the ROBINS‐I tool (Sterne et al., 2016), generally presenting a moderate risk of bias, particularly due to confounding factors. However, biases in participant selection, intervention classification, outcome measurement and result reporting were largely considered low (Table S3).

### 3.4 Mortality

**FIGURE 2:**
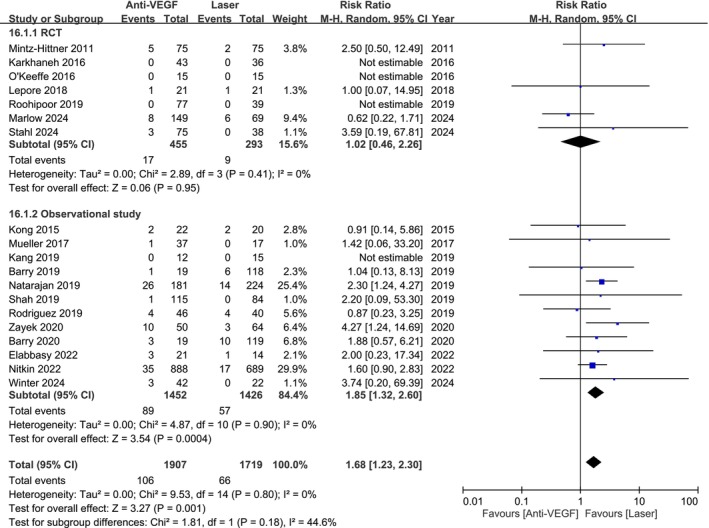
Forest plot of the mortality rate comparing anti‐VEGF and laser groups. CI, confidence interval.

Our meta‐analysis of 3626 individuals from 7 RCTs and 12 observational studies found a higher mortality risk with anti‐VEGF treatment compared to laser therapy (RR: 1.68; 95% CI: 1.23–2.30; Figure 2). Publication bias was assessed using a funnel plot (Figure S1). In observational studies, anti‐VEGF therapy showed a significantly higher mortality risk (RR 1.85; 95% CI 1.32–2.60). However, the RCT subgroup showed no significant increase (RR 1.02; 95% CI 0.46–2.26).

Several studies have reported causes of death across various organ systems, with respiratory diseases being the leading cause in both treatment groups. The causes and their respective proportions are detailed in Figure S2.

### 3.5 Retinal detachment

**FIGURE 3:**
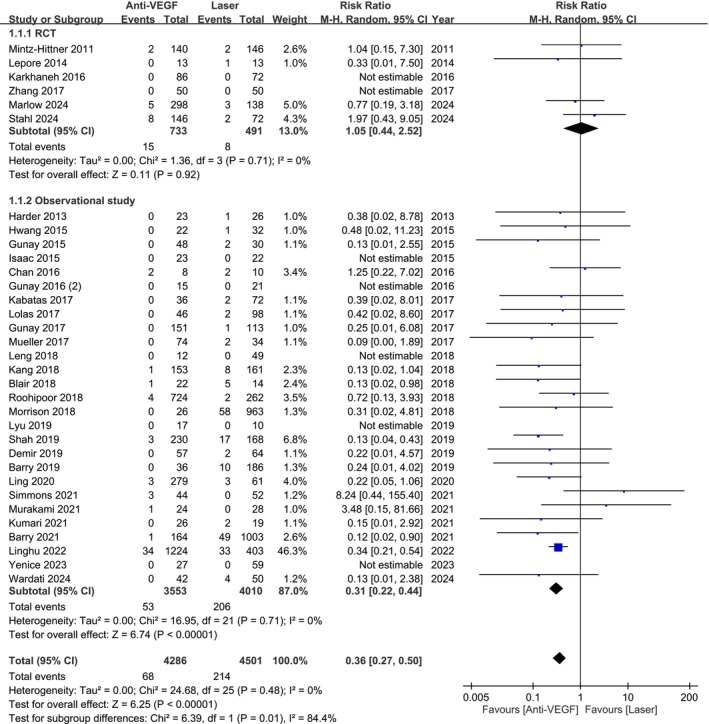
Forest plot of the retinal detachment rate comparing anti‐VEGF and laser groups. CI, confidence interval.

Our analysis of 8787 eyes from 6 RCTs and 27 observational studies found a significantly lower risk of retinal detachment with anti‐VEGF treatment compared to laser therapy (RR 0.36; 95% CI 0.27–0.50; Figure 3). Subgroup analysis showed no significant difference in RCTs (RR 1.05; 95% CI 0.44–2.52), while observational studies reported a lower incidence with anti‐VEGF (RR 0.31; 95% CI 0.22–0.44).

### 3.6 Vitrectomy/scleral buckle/surgery

**FIGURE 4:**
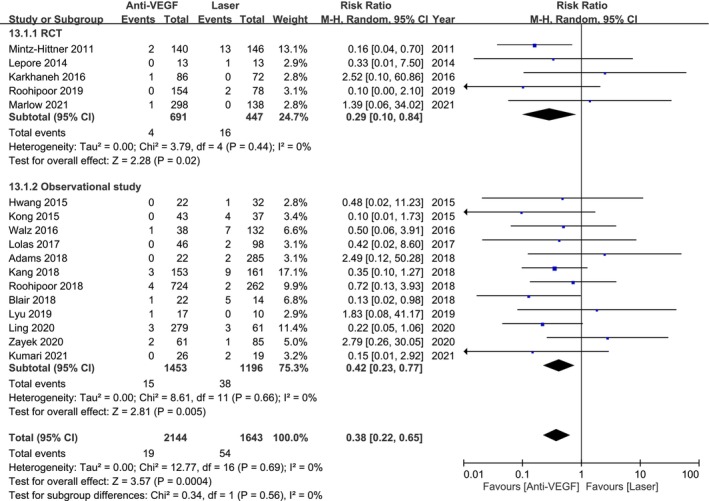
Forest plot of the surgical rate comparing anti‐VEGF and laser groups. CI, confidence interval.

Analysis of 3787 eyes from 5 RCTs and 12 observational studies showed that anti‐VEGF therapy significantly reduced the need for surgical interventions in infants with ROP (RR 0.38; 95% CI 0.22–0.65; Figure 4). Subgroup analysis showed a reduction in surgical interventions with anti‐VEGF therapy in RCTs (RR 0.29; 95% CI 0.10–0.84) and observational studies (RR 0.42; 95% CI 0.23–0.77).

### 3.7 Myopia

**FIGURE 5:**
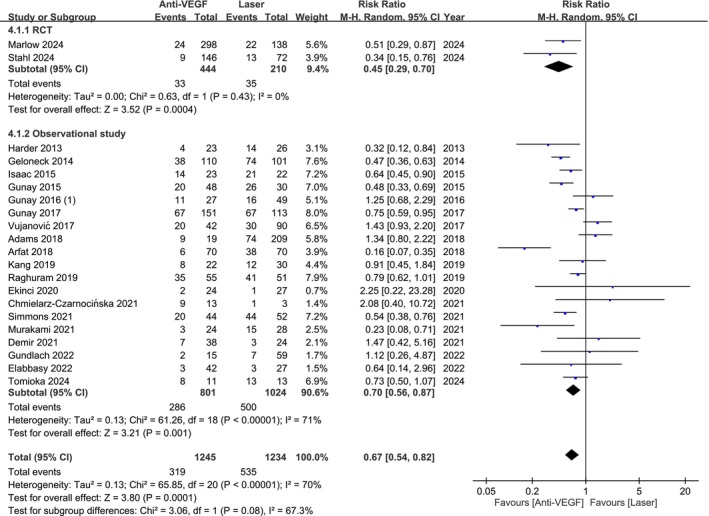
Forest plot of the myopia rate comparing anti‐VEGF and laser groups. CI, confidence interval.

Analysis of 2479 eyes from 2 RCTs and 19 observational studies showed a lower incidence of myopia with anti‐VEGF therapy than laser (RR 0.67; 95% CI 0.54–0.82; Figure 5). Subgroup analysis also indicated reduced risk in both RCTs (RR 0.45; 95% CI 0.29–0.70) and observational studies (RR 0.70; 95% CI 0.56–0.87).

### 3.8 Developmental delay/cerebral palsy

**FIGURE 6:**
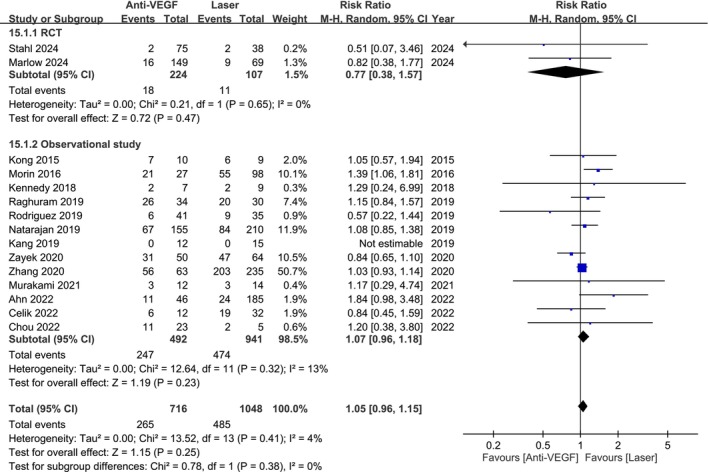
Forest plot of the incidence of developmental delay or cerebral palsy comparing anti‐VEGF and laser groups. CI, confidence interval.

Analysis of 1764 patients from 2 RCTs and 13 observational studies found no significant difference in developmental delay or cerebral palsy risk between anti‐VEGF and laser therapy (RR 1.05; 95% CI 0.96–1.15; Figure 6). Subgroup analysis also demonstrated no significant difference in both RCTs (RR 0.77; 95% CI 0.38–1.57) and observational studies (RR 1.07; 95% CI 0.96–1.18).

### 3.9 Retina dragging/macular dragging/macular traction

Analysis of 4455 eyes showed anti‐VEGF therapy significantly reduced the risk of retinal dragging, macular dragging or macular traction compared to laser therapy (RR 0.33; 95% CI 0.18–0.59; Figure S3). Subgroup analysis confirmed reduced risk in both RCTs (RR 0.24; 95% CI 0.06–0.96) and observational studies (RR 0.37; 95% CI 0.17–0.78).

### 3.10 Retinal holes

An observational study of 52 eyes found no significant difference between treatments (RR 5.80; 95% CI 0.29–115.21; Figure S4).

### 3.11 Macular fold/retinal fold

Data from 2351 eyes showed no significant difference between treatments (RR 1.85; 95% CI 0.26–13.07; Figure S5), with RCTs (RR 0.65; 95% CI 0.12–3.63) and observational studies (RR 9.88; 95% CI 0.24–404.10) yielding similar results.

### 3.12 Macular Ectopia

Analysis of 625 eyes showed no significant difference in the risk of macular ectopia (RR 0.37; 95% CI 0.12–1.16; Figure S6), with similar findings in RCTs (RR 0.46; 95% CI 0.12–1.82) and observational studies (RR 0.22; 95% CI 0.03–1.74).

### 3.13 Haemorrhage

Data from 3974 eyes across 4 RCTs and 13 observational studies showed no significant difference in the risk of haemorrhage (RR 1.01; 95% CI 0.57–1.76; Figure S7). Subgroup analysis found no significant difference in RCTs (RR 0.88; 95% CI 0.26–3.00) or observational studies (RR 1.14; 95% CI 0.58–2.23).

### 3.14 Uveitis

One observational study of 46 eyes found no significant difference (RR 0.07; 95% CI 0.00–1.45; Figure S8).

### 3.15 Endophthalmitis

The overall analysis of 1845 eyes found no significant difference in risk between anti‐VEGF and laser treatments (RR 1.39; 95% CI 0.06–34.02; Figure S9).

### 3.16 Cataract/lens opacity

Data from 5391 eyes across 6 RCTs and 13 observational studies showed no significant difference in the risk of cataract or lens opacity (RR 1.47; 95% CI 0.61–3.55; Figure S10). Subgroup analysis found no significant difference in RCTs (RR 1.26; 95% CI 0.28–5.66) or observational studies (RR 1.59; 95% CI 0.53–4.74).

### 3.17 Keratitis

One observational study of 108 eyes found no significant difference (RR 1.40; 95% CI 0.06–33.51; Figure S11).

### 3.18 Corneal erosion

Analysis of 1123 eyes from 4 observational studies found no significant difference (RR 0.90; 95% CI 0.25–3.29; Figure S12).

### 3.19 Corneal opacity

Data from 3053 eyes showed no significant difference (RR 0.79; 95% CI 0.20–3.07; Figure S13). Subgroup analysis found no significant difference in RCTs (RR 0.17; 95% CI 0.02–1.56) or observational studies (RR 2.02; 95% CI 0.36–11.47).

## 4 DISCUSSION

VEGF regulation in angiogenesis has been a focal point in understanding the pathophysiology of various diseases. Early studies on intravitreal anti‐VEGF injections mainly explored the potential risks, such as arterial thromboembolic events and significant cardiovascular or nonocular hemorrhagic events, yet found no substantial connections (Cheng et al., 2012; Thulliez et al., 2014). However, later research indicated an increased risk of vascular events in patients receiving regular anti‐VEGF treatment (Avery & Gordon, 2016; Ueta et al., 2014).

When examining mortality rates, the picture is more complex. Avery et al. (2016) proposed that monthly anti‐VEGF injections might increase mortality in diabetic macular oedema patients, though other meta‐analyses found no significant rise in mortality across a variety of retinal and macular conditions (Dalvin et al., 2019; Reibaldi et al., 2020; Ueta et al., 2014).

Research on anti‐VEGF in infants with ROP remains relatively scarce. Natarajan et al. (2019) found no mortality difference between intravitreal bevacizumab (IVB) and laser therapy during hospitalization, but follow‐up at 18–26 months' corrected age showed higher mortality in the IVB group. Zayek et al. (2020) reported higher initial mortality in IVB‐treated infants (16%) versus laser‐treated (3%), though this was not statistically significant after adjustment for confounders. Moreover, two meta‐analyses of RCTs concluded that anti‐VEGF and laser therapy had comparable mortality rates in ROP infants (Sankar et al., 2018; Taher et al., 2022).

Frieden (2017) argued that while RCTs minimize bias and confounding, their small sample sizes and short follow‐up periods can limit the assessment of rare but severe adverse effects. Therefore, employing additional research data, such as from observational studies, can provide a broader perspective for informed clinical decisions.

Given the rarity and severity of adverse effects like mortality, we included both RCTs and observational studies for a more comprehensive analysis. While observational studies are valuable for identifying rare safety events and allowing long‐term follow‐up, our subgroup analysis revealed inconsistencies between observational and RCT findings, suggesting the potential influence of confounding factors. Notably, the RCT subgroup showed no significant difference in mortality. As a result, we emphasize the need for large‐scale RCTs with extended follow‐up periods to better evaluate treatment safety and resolve discrepancies between study designs.

To conduct a comprehensive analysis, we included mortality from all causes for infants undergoing either anti‐VEGF or laser treatment. Moreover, according to the methodological approach proposed by Cheng et al. (2016), when analysing negative outcomes such as death rates, studies with ‘both‐armed zero‐event’ cases should be removed to reduce assessment bias. Thus, studies reporting no deaths in either treatment group were excluded from the overall effect synthesis.

When exploring the causes of mortality, while anti‐VEGF therapy in adults has been linked to vascular adverse events (Avery & Gordon, 2016; Ueta et al., 2014), our study indicates that in infants, respiratory diseases are the primary cause of mortality in both treatment groups, with a notably higher incidence in those treated with anti‐VEGF (Figure S2).

VEGF plays a critical role in lung development, and its reduced expression has been associated with abnormal lung growth and bronchopulmonary dysplasia (Bhatt et al., 2001; Mariduena et al., 2022; Myint et al., 2021). Additionally, Khalili et al. (2018) demonstrated in animal models that anti‐VEGF antibodies primarily affect the lungs, potentially leading to pulmonary hypertension. Nitkin et al. (2022) further found that patients receiving anti‐VEGF therapy required pulmonary vasodilators more often than those treated with laser therapy, suggesting a possible link to pulmonary hypertension. These findings may help explain the increased mortality from respiratory diseases observed in our analysis.

Existing meta‐analyses show conflicting results on retinal detachment risk. Popovic et al. (2021) found no significant difference between anti‐VEGF and laser therapy, while Chen et al. (2023), in a larger study, reported a higher risk with laser treatment. Our comprehensive analysis, which included 6 RCTs and 27 observational studies, demonstrated a considerably increased risk of retinal detachment among patients treated with laser therapy.

Popovic et al. (2021) also reported that anti‐VEGF therapy reduces the need for surgical interventions. Our research consistently revealed that infants with ROP who received anti‐VEGF treatment were less likely to require subsequent surgical procedures compared to those treated with laser therapy.

In terms of refractive outcomes, Popovic et al. (2021) found no difference in refractive outcomes between anti‐VEGF and laser therapy, suggesting that ROP severity plays a larger role in myopia development. However, numerous meta‐analyses have reported lower rates of myopia in eyes treated with anti‐VEGF compared to laser (Chen et al., 2023; Kong et al., 2021; Li et al., 2018; Tan et al., 2019; Wang et al., 2020). Our meta‐analysis of 21 studies aligns with these findings, demonstrating a lower risk of myopia following anti‐VEGF therapy compared to laser.

As VEGF is a vital factor for angiogenesis across various organs, and the same isoform of VEGF has been detected in the brain and eyes in animal experiments (Ng et al., 2001), concerns have arisen about whether using anti‐VEGF to treat ROP might also affect nervous system development. Sato et al. (2012) discovered that IVB therapy could lower systemic VEGF levels in infants. Also, research has shown that anti‐VEGF agents can remain in circulation for up to 2 months, during which time serum VEGF levels decrease and take 2–3 months to normalize (Kong, Bhatt, et al. (2015); Huang et al., 2018; Cheng et al., 2020). On the other hand, Kong, Bhatt, et al. (2015) noted that laser treatment also lowers serum VEGF levels, likely due to retinal cell destruction, though its effects are less pronounced than those of IVB therapy.

Meta‐analyses on IVB therapy show conflicting results, with one finding no increased risk of severe neurodevelopmental issues and another linking it to higher cognitive impairment risk (Kaushal et al., 2021; Tsai et al., 2021). Studies in 18‐month‐old ROP infants reported higher risks of severe neurodevelopmental disabilities and delays in language and social skills with IVB compared to laser (Arima et al., 2020; Morin et al., 2016). However, growing evidence suggests that there may be no significant difference in neurodevelopmental outcomes between ROP patients treated with anti‐VEGF or laser (Ahn et al., 2022; Celik et al., 2022; Chen et al., 2018; Chou et al., 2022; Kennedy et al., 2018; Marlow et al., 2021; Murakami et al., 2021; Natarajan et al., 2019; Raghuram et al., 2019; Rodriguez et al., 2019; Zayek et al., 2020; Zhang et al., 2020). Follow‐up was typically under 28 months, with two studies extending to 5 years, still showing no significant cognitive or developmental differences (Chou et al., 2022; Murakami et al., 2021).

While a meta‐analysis by Diggikar et al. (2023) linked anti‐VEGF therapy to a higher risk of cognitive impairment compared to laser treatment, our analysis found no significant difference in neurodevelopmental impairment or cerebral palsy between treatments.

### 4.1 Strengths and limitations

Our meta‐analysis has several strengths. We included a large‐scale sample from both RCTs and observational studies. We comprehensively assessed multiple outcomes, exceeding previous meta‐analyses, and observed low heterogeneity among studies. Additionally, we conducted a subgroup analysis of RCTs and observational studies. While our initial analysis suggested a higher mortality risk with anti‐VEGF therapy, the RCTs subgroup showed no significant difference in mortality risk, highlighting the potential influence of confounding variables in observational studies. We also explored the causes of death and found that respiratory diseases were the leading cause of mortality, with a higher proportion in infants receiving anti‐VEGF therapy. While previous studies on neurodevelopmental outcomes have reported conflicting results, our analysis provides clear evidence, showing no significant differences in neurodevelopmental outcomes between the two treatments.

This study has limitations. First, we did not analyse the effects of different anti‐VEGF dosages, frequencies or specific agents, limiting insights into their individual efficacy and safety. Notably, combining all anti‐VEGF agents in analysis may mask important differences in systemic effects. Intravitreal injections of bevacizumab, aflibercept and conbercept have been shown to reduce serum VEGF levels (Cheng et al., 2020; Huang et al., 2018; Kong, Bhatt, et al., 2015; Sato et al., 2012), whereas the RAINBOW trial (Stahl et al., 2019) found no clear evidence of serum VEGF suppression following intravitreal ranibizumab. Therefore, it is possible that ranibizumab may be less likely to cause systemic adverse effects associated with VEGF suppression than other anti‐VEGF agents.

Second, selection bias may affect non‐randomized studies. Findings from the BEAT‐ROP trial (Mintz‐Hittner et al., 2011), which demonstrated a benefit of bevacizumab over laser therapy in Zone I but not in Zone II disease, may have led clinicians to preferentially use anti‐VEGF therapy for more severe ROP or less mature infants. Conversely, laser therapy may be more frequently applied in more mature eyes or infants. This hypothesis may help explain why a higher mortality risk associated with anti‐VEGF therapy was observed in the observational studies but not in RCTs.

Third, the cause of high heterogeneity in myopia outcomes was not explored. Finally, the included studies lack RCTs with long‐term follow‐up, resulting in insufficient analysis of long‐term treatment outcomes. To address these limitations, future large‐scale RCTs with extended follow‐up are needed for a more comprehensive comparison of bevacizumab, ranibizumab, aflibercept and laser therapies.

## 5 CONCLUSIONS

Our meta‐analysis shows that anti‐VEGF therapy reduces retinal detachment, surgical interventions, and myopia risk in infants with ROP without increasing neurodevelopmental impairment or cerebral palsy. Notably, we identified a higher mortality risk associated with anti‐VEGF treatment, a concern not widely reported in previous studies. However, in the subgroup analysis, the RCT subgroup showed no significant difference in mortality risk, suggesting that confounding factors in observational studies may have influenced the results. This highlights the need for large, well‐controlled trials that adjust for confounders to further evaluate the safety of anti‐VEGF therapy.

## AUTHOR CONTRIBUTIONS

Conception and design: W.D.W, Y.H.Y, C.H.L, S.H.H, T.H.T, Y.G.C, D.W.L, J.T.C, K.H.C, S.I.P, W.T.Y; Analysis and interpretation: W.D.W, Y.H.Y, C.H.L, S.H.H, T.H.T, Y.G.C, W.T.Y; Data collection: W.D.W, W.T.Y; Obtained funding: S.I.P, W.T.Y; Overall responsibility: W.D.W, Y.H.Y, C.H.L, S.H.H, T.H.T, Y.G.C, D.W.L, J.T.C, K.H.C, S.I.P, W.T.Y.

## FUNDING INFORMATION

This study was funded by a grant from the Tri‐Service General Hospital Research Fund (TSGH_D_114184). The funder had no role in the design and conduct of the study.

## Supporting information


Appendix S1.

## Data Availability

All the data used in this study are available within the article. No additional data is available.

## References

[aos17541-bib-0001] Adams, G.G. , Bunce, C. , Xing, W. , Butler, L. , Long, V. , Reddy, A. et al. (2018) Retinopathy of prematurity in the United Kingdom: retreatment rates, visual and structural 1‐year outcomes. Eye, 32(11), 1752–1759. 30013158 10.1038/s41433-018-0151-y PMC6224459

[aos17541-bib-0002] Ahn, J.H. , Lee, K.M. , Kim, M.J. , Park, H.‐K. , Kim, Y.J. , Ahn, S.J. et al. (2022) Neurodevelopmental outcomes in very low birthweight infants with retinopathy of prematurity in a nationwide cohort study. Scientific Reports, 12(1), 5053. 35322163 10.1038/s41598-022-09053-8 PMC8943194

[aos17541-bib-0003] Alon, T. , Hemo, I. , Itin, A. , Pe'er, J. , Stone, J. & Keshet, E. (1995) Vascular endothelial growth factor acts as a survival factor for newly formed retinal vessels and has implications for retinopathy of prematurity. Nature Medicine, 1(10), 1024–1028. 10.1038/nm1095-1024 7489357

[aos17541-bib-0004] Arfat, M. (2018) Comparison of complications of intravitreal bevacizumab with laser photocoagulation for the treatment of retinopathy of pre‐maturity. Pakistan Journal of Medical and Health Sciences, 12, 1079–1088.

[aos17541-bib-0005] Arima, M. , Akiyama, M. , Fujiwara, K. , Mori, Y. , Inoue, H. , Seki, E. et al. (2020) Neurodevelopmental outcomes following intravitreal bevacizumab injection in Japanese preterm infants with type 1 retinopathy of prematurity. PLoS One, 15(3), e0230678. 32196539 10.1371/journal.pone.0230678 PMC7083318

[aos17541-bib-0006] Avery, R.L. & Gordon, G.M. (2016) Systemic safety of prolonged monthly anti‐vascular endothelial growth factor therapy for diabetic macular edema: a systematic review and meta‐analysis. JAMA Ophthalmology, 134(1), 21–29. 26513684 10.1001/jamaophthalmol.2015.4070

[aos17541-bib-0007] Barry, G.P. , Tauber, K.A. , Fisher, M. , Greenberg, S. , Zobal‐Ratner, J. & Binenbaum, G. (2019) Short‐term retinal detachment risk after treatment of type 1 retinopathy of prematurity with laser photocoagulation versus intravitreal bevacizumab. Journal of AAPOS, 23(5), 260.e1–260.e4. 10.1016/j.jaapos.2019.05.013 31513902

[aos17541-bib-0008] Barry, G.P. , Tauber, K.A. , Greenberg, S. , Lajoie, J. , Afroze, F. , Oechsner, H. et al. (2020) A comparison of respiratory outcomes after treating retinopathy of prematurity with laser photocoagulation or intravitreal bevacizumab. Ophthalmology Retina, 4(12), 1202–1208. 32512055 10.1016/j.oret.2020.06.002

[aos17541-bib-0009] Barry, G.P. , Yu, Y. , Ying, G.‐S. , Tomlinson, L.A. , Lajoie, J. , Fisher, M. et al. (2021) Retinal detachment after treatment of retinopathy of prematurity with laser versus intravitreal anti‐vascular endothelial growth factor. Ophthalmology, 128(8), 1188–1196. 33387554 10.1016/j.ophtha.2020.12.028 PMC8819483

[aos17541-bib-0010] Bhatt, A.J. , Pryhuber, G.S. , Huyck, H. , Watkins, R.H. , Metlay, L.A. & Maniscalco, W.M. (2001) Disrupted pulmonary vasculature and decreased vascular endothelial growth factor, Flt‐1, and TIE‐2 in human infants dying with bronchopulmonary dysplasia. American Journal of Respiratory and Critical Care Medicine, 164(10 Pt 1), 1971–1980. 11734454 10.1164/ajrccm.164.10.2101140

[aos17541-bib-0011] Blair, M. , Gonzalez, J.M.G. , Snyder, L. , Schechet, S. , Greenwald, M. , Shapiro, M. et al. (2018) Bevacizumab or laser for aggressive posterior retinopathy of prematurity. Taiwan Journal of Ophthalmology, 8(4), 243–248. 30637196 10.4103/tjo.tjo_69_18 PMC6302568

[aos17541-bib-0012] Celik, P. , Ayranci Sucakli, I. , Kara, C. , Petricli, I.S. , Kavurt, S. , Celik, I.H. et al. (2022) Bevacizumab and neurodevelopmental outcomes of preterm infants with retinopathy of prematurity: should we still worry? The Journal of Maternal‐Fetal & Neonatal Medicine, 35(3), 415–422. 33618591 10.1080/14767058.2021.1888913

[aos17541-bib-0013] Chan, J.J.T. , Lam, C.P.S. , Kwok, M.K.M. , Wong, R.L.M. , Lee, G.K.Y. , Lau, W.W.Y. et al. (2016) Risk of recurrence of retinopathy of prematurity after initial intravitreal ranibizumab therapy. Scientific Reports, 6(1), 27082. 27256987 10.1038/srep27082 PMC4891718

[aos17541-bib-0014] Chen, J. , Hao, Q. , Zhang, J. , Du, Y. , Chen, H. & Cheng, X. (2023) The efficacy and ocular safety following aflibercept, conbercept, ranibizumab, bevacizumab, and laser for retinopathy of prematurity: a systematic review and meta‐analysis. Italian Journal of Pediatrics, 49(1), 136. 37814332 10.1186/s13052-023-01543-3 PMC10561404

[aos17541-bib-0015] Chen, T.A. , Schachar, I.H. & Moshfeghi, D. (2018) Outcomes of intravitreal bevacizumab and diode laser photocoagulation for treatment‐warranted retinopathy of prematurity. Ophthalmic Surgery, Lasers & Imaging Retina, 49, 126–131. 10.3928/23258160-20180129-07 29443362

[aos17541-bib-0016] Cheng, J. , Pullenayegum, E. , Marshall, J.K. , Iorio, A. & Thabane, L. (2016) Impact of including or excluding both‐armed zero‐event studies on using standard meta‐analysis methods for rare event outcome: a simulation study. BMJ Open, 6(8), e010983. 10.1136/bmjopen-2015-010983 PMC5013416 27531725

[aos17541-bib-0017] Cheng, J.W. , Cheng, S.W. , Lu, G.C. & Wei, R.L. (2012) Effect of intravitreal anti‐vascular endothelial growth factor therapy on the risk of arterial thromboembolic events: a meta‐analysis. PLoS One, 7(7), e41325. 22829940 10.1371/journal.pone.0041325 PMC3400599

[aos17541-bib-0018] Cheng, Y. , Zhu, X. , Linghu, D. , Xu, Y. & Liang, J. (2020) Serum levels of cytokines in infants treated with conbercept for retinopathy of prematurity. Scientific Reports, 10(1), 12695. 32728160 10.1038/s41598-020-69684-7 PMC7391743

[aos17541-bib-0019] Chmielarz‐Czarnocińska, A. , Pawlak, M. , Rzeszotarska, A. , Szpecht, D. , Szymankiewicz‐Bręborowicz, M. & Gotz‐Więckowska, A. (2021) Anatomical and functional outcomes of treatment for retinopathy of prematurity (ROP) in zone I. Klinika Oczna, 123(4), 203–209.

[aos17541-bib-0020] Chou, H.D. , Shih, C.P. , Huang, Y.S. , Liu, L. , Lai, C.C. & Chen, K.J. (2022) Cognitive outcomes following intravitreal bevacizumab for retinopathy of prematurity: 4‐to 6‐year outcomes in a prospective cohort. American Journal of Ophthalmology, 234, 59–70. 34283975 10.1016/j.ajo.2021.06.034

[aos17541-bib-0021] Cunningham, E.T., Jr. , Adamis, A.P. , Altaweel, M. , Aiello, L.P. , Bressler, N.M. , D'Amico, D.J. et al. (2005) A phase II randomized double‐masked trial of pegaptanib, an anti‐vascular endothelial growth factor aptamer, for diabetic macular edema. Ophthalmology, 112(10), 1747–1757. 16154196 10.1016/j.ophtha.2005.06.007

[aos17541-bib-0022] Dalvin, L.A. , Starr, M.R. , AbouChehade, J.E. , Damento, G.M. , Garcia, M. , Shah, S.M. et al. (2019) Association of intravitreal anti‐vascular endothelial growth factor therapy with risk of stroke, myocardial infarction, and death in patients with exudative age‐related macular degeneration. JAMA Ophthalmology, 137(5), 483–490. 30703203 10.1001/jamaophthalmol.2018.6891 PMC6512306

[aos17541-bib-0023] Demir, S.T. , Güven, D. , Karapapak, M. , Uslu, H.S. , Bülbül, A. , Türker, İ.Ç. et al. (2019) Evaluation of treatment models in the treatment of retinopathy of prematurity. Sisli Etfal Hastanesi Tip Bülteni, 53(3), 290–295. 32377098 10.14744/SEMB.2018.60465 PMC7192269

[aos17541-bib-0024] Demir, S.T. , Yesiltas, S.K. , Karapapak, M. , Ozyurek, E. , Bulbul, A. & Uslu, H.S. (2021) Evaluation of the effect of different treatment management on refractive outcomes in severe retinopathy of prematurity. Sisli Etfal Hastanesi Tip Bülteni, 55(4), 545–550. 35317380 10.14744/SEMB.2021.34966 PMC8907699

[aos17541-bib-0025] Diggikar, S. , Gurumoorthy, P. , Trif, P. , Mudura, D. , Nagesh, N.K. , Galis, R. et al. (2023) Retinopathy of prematurity and neurodevelopmental outcomes in preterm infants: a systematic review and meta‐analysis. Frontiers in Pediatrics, 11, 1055813. 37009271 10.3389/fped.2023.1055813 PMC10050340

[aos17541-bib-0026] Ekinci, D.Y. & Çelik, K. (2020) Comparison of the efficacy between intravitreal aflibercept and laser photocoagulation in the treatment of retinopathy of prematurity. Journal of Pediatric Ophthalmology and Strabismus, 57(1), 54–60. 31972042 10.3928/01913913-20191104-01

[aos17541-bib-0027] Elabbasy, A. , Abdelbaky, M. , Al‐Shehri, H. , Padua, I. , Hamed, A. , Kashlan, A. et al. (2022) Comparative analysis of intravitreal ranibizumab versus laser therapy for retinopathy of prematurity. Archives of Pharmacy Practice, 13(2), 30–36.

[aos17541-bib-0028] Frieden, T.R. (2017) Evidence for health decision making — beyond randomized, controlled trials. The New England Journal of Medicine, 377(5), 465–475. 28767357 10.1056/NEJMra1614394

[aos17541-bib-0029] Geloneck, M.M. , Chuang, A.Z. , Clark, W.L. , Hunt, M.G. , Norman, A.A. , Packwood, E.A. et al. (2014) Refractive outcomes following bevacizumab monotherapy compared with conventional laser treatment: a randomized clinical trial. JAMA Ophthalmology, 132(11), 1327–1333. 25103848 10.1001/jamaophthalmol.2014.2772

[aos17541-bib-0030] Good, W.V. & Early Treatment for Retinopathy of Prematurity Cooperative Group . (2004) Final results of the early treatment for retinopathy of prematurity (ETROP) randomized trial. Transactions of the American Ophthalmological Society, 102, 233–248; discussion 248–50. 15747762 PMC1280104

[aos17541-bib-0031] Good, W.V. & Early Treatment for Retinopathy of Prematurity Cooperative Group . (2006) The early treatment for retinopathy of prematurity study: structural findings at age 2 years. British Journal of Ophthalmology, 90(11), 1378–1382. 16914473 10.1136/bjo.2006.098582 PMC1857487

[aos17541-bib-0032] Good, W.V. , Hardy, R.J. , Dobson, V. , Palmer, E.A. , Phelps, D.L. , Quintos, M. et al. (2003) Revised indications for the treatment of retinopathy of prematurity: results of the early treatment for retinopathy of prematurity randomized trial: results of the early treatment for retinopathy of prematurity randomized trial. Archives of Ophthalmology, 121(12), 1684–1694. 14662586 10.1001/archopht.121.12.1684

[aos17541-bib-0033] Gragoudas, E.S. , Adamis, A.P. , Cunningham, E.T., Jr. , Feinsod, M. , Guyer, D.R. & VEGF Inhibition Study in Ocular Neovascularization Clinical Trial Group . (2004) Pegaptanib for neovascular age‐related macular degeneration. New England Journal of Medicine, 351(27), 2805–2816. 15625332 10.1056/NEJMoa042760

[aos17541-bib-0034] Gunay, M. , Celik, G. , Gunay, B.O. , Aktas, A. , Karatekin, G. & Ovali, F. (2015) Evaluation of 2‐year outcomes following intravitreal bevacizumab (IVB) for aggressive posterior retinopathy of prematurity. Arquivos Brasileiros de Oftalmologia, 78(5), 300–304. 26466229 10.5935/0004-2749.20150079

[aos17541-bib-0035] Gunay, M. , Celik, G. , Tuten, A. , Karatekin, G. , Bardak, H. & Ovali, F. (2016) Characteristics of severe retinopathy of prematurity in infants with birth weight above 1500 grams at a referral center in Turkey. PLoS One, 11(8), e0161692. 27548628 10.1371/journal.pone.0161692 PMC4993354

[aos17541-bib-0036] Gunay, M. , Sekeroglu, M.A. , Bardak, H. , Celik, G. , Esenulku, C.M. , Hekimoglu, E. et al. (2016) Evaluation of refractive errors and ocular biometric outcomes after intravitreal bevacizumab for retinopathy of prematurity. Strabismus, 24(2), 84–88. 27120579 10.3109/09273972.2016.1159232

[aos17541-bib-0037] Gunay, M. , Sukgen, E.A. , Celik, G. & Kocluk, Y. (2017) Comparison of bevacizumab, ranibizumab, and laser photocoagulation in the treatment of retinopathy of prematurity in Turkey. Current Eye Research, 42(3), 462–469. 27420302 10.1080/02713683.2016.1196709

[aos17541-bib-0038] Gundlach, B.S. , Kokhanov, A. , Altendahl, M. , Suh, S.Y. , Fung, S. , Demer, J. et al. (2022) Real‐world visual outcomes of laser and anti‐VEGF treatments for retinopathy of prematurity. American Journal of Ophthalmology, 238, 86–96. 34788594 10.1016/j.ajo.2021.11.015

[aos17541-bib-0039] Harder, B.C. , Schlichtenbrede, F.C. , von Baltz, S. , Jendritza, W. , Jendritza, B. & Jonas, J.B. (2013) Intravitreal bevacizumab for retinopathy of prematurity: refractive error results. American Journal of Ophthalmology, 155(6), 1119–1124.e1. 23490192 10.1016/j.ajo.2013.01.014

[aos17541-bib-0040] Higgins, J. , Thomas, J. , Chandler, J. , Cumpston, M. , Li, T. , Page, M.J. et al. (2024) Cochrane Handbook for Systematic Reviews of Interventions version 6. Cochrane. 10.1002/14651858.ED000142 PMC10284251 31643080

[aos17541-bib-0041] Huang, C.Y. , Lien, R. , Wang, N.K. , Chao, A.N. , Chen, K.J. , Chen, T.L. et al. (2018) Changes in systemic vascular endothelial growth factor levels after intravitreal injection of aflibercept in infants with retinopathy of prematurity. Graefe's Archive for Clinical and Experimental Ophthalmology, 256(3), 479–487. 10.1007/s00417-017-3878-4 29290015

[aos17541-bib-0042] Hwang, C.K. , Hubbard, G.B. , Hutchinson, A.K. & Lambert, S.R. (2015) Outcomes after intravitreal bevacizumab versus laser photocoagulation for retinopathy of prematurity: a 5‐year retrospective analysis. Ophthalmology, 122(5), 1008–1015. 25687024 10.1016/j.ophtha.2014.12.017 PMC4414677

[aos17541-bib-0043] Imamoglu, E.Y. , Gunay, M. , Gursoy, T. , Imamoglu, S. , Ekmekci, O.B. , Celik, G. et al. (2014) Effect of laser photocoagulation on plasma levels of VEGF‐A, VEGFR‐2, and Tie2 in infants with retinopathy of prematurity. Journal of the American Association for Pediatric Ophthalmology and Strabismus, 18(5), 466–470. 10.1016/j.jaapos.2014.07.159 25266828

[aos17541-bib-0044] Isaac, M. , Mireskandari, K. & Tehrani, N. (2015) Treatment of type 1 retinopathy of prematurity with bevacizumab versus laser. Journal of AAPOS, 19(2), 140–144. 25892041 10.1016/j.jaapos.2015.01.009

[aos17541-bib-0045] Kabataş, E.U. , Kurtul, B.E. , Altıaylık Özer, P. & Kabataş, N. (2017) Comparison of intravitreal bevacizumab, intravitreal ranibizumab and laser photocoagulation for treatment of type 1 retinopathy of prematurity in Turkish preterm children. Current Eye Research, 42(7), 1054–1058. 28128986 10.1080/02713683.2016.1264607

[aos17541-bib-0046] Kang, H.G. , Choi, E.Y. , Byeon, S.H. , Kim, S.S. , Koh, H.J. , Lee, S.C. et al. (2018) Intravitreal ranibizumab versus laser photocoagulation for retinopathy of prematurity: efficacy, anatomical outcomes and safety. British Journal of Ophthalmology, 103(9), 1332–1336. 30514709 10.1136/bjophthalmol-2018-312272

[aos17541-bib-0047] Kang, H.G. , Kim, T.Y. , Han, J. & Han, S.H. (2019) Refractive outcomes of 4‐year‐old children after intravitreal anti‐vascular endothelial growth factor versus laser photocoagulation for retinopathy of prematurity. Korean Journal of Ophthalmology, 33(3), 272–278. 31179659 10.3341/kjo.2019.0011 PMC6557791

[aos17541-bib-0048] Karkhaneh, R. , Khodabande, A. , Riazi‐Eafahani, M. , Roohipoor, R. , Ghassemi, F. , Imani, M. et al. (2016) Efficacy of intravitreal bevacizumab for zone‐II retinopathy of prematurity. Acta Ophthalmologica, 94(6), e417–e420. 27009449 10.1111/aos.13008

[aos17541-bib-0049] Kaushal, M. , Razak, A. , Patel, W. , Pullattayil, A.K. & Kaushal, A. (2021) Neurodevelopmental outcomes following bevacizumab treatment for retinopathy of prematurity: a systematic review and meta‐analysis. Journal of Perinatology, 41(6), 1225–1235. 33293666 10.1038/s41372-020-00884-9

[aos17541-bib-0050] Kennedy, K.A. , Mintz‐Hittner, H.A. & BEAT‐ROP Cooperative Group . (2018) Medical and developmental outcomes of bevacizumab versus laser for retinopathy of prematurity. Journal of the American Association for Pediatric Ophthalmology and Strabismus, 22(1), 61–65.e1. 10.1016/j.jaapos.2017.10.006 PMC5826862 29223789

[aos17541-bib-0051] Khalili, S. , Shifrin, Y. , Pan, J. , Belik, J. & Mireskandari, K. (2018) The effect of a single anti‐vascular endothelial growth factor injection on neonatal growth and organ development: in‐vivo study. Experimental Eye Research, 169, 54–59. 29421328 10.1016/j.exer.2018.01.020

[aos17541-bib-0052] Kim, S.J. , Port, A.D. , Swan, R. , Campbell, J.P. , Chan, R.V.P. & Chiang, M.F. (2018) Retinopathy of prematurity: a review of risk factors and their clinical significance. Survey of Ophthalmology, 63(5), 618–637. 29679617 10.1016/j.survophthal.2018.04.002 PMC6089661

[aos17541-bib-0053] Kong, L. , Bhatt, A.R. , Demny, A.B. , Coats, D.K. , Li, A. , Rahman, E.Z. et al. (2015) Pharmacokinetics of bevacizumab and its effects on serum VEGF and IGF‐1 in infants with retinopathy of prematurity. Investigative Ophthalmology & Visual Science, 56(2), 956–961. 25613938 10.1167/iovs.14-15842

[aos17541-bib-0054] Kong, L. , Dinh, K. , Schechet, S. , Coats, D. , Voigt, R. , Demny, A. et al. (2015) Comparison of ocular and developmental outcomes in laser‐and bevacizumab‐treated infants with retinopathy of prematurity. Ophthalmology Research: An International Journal, 3(1), 13–22.

[aos17541-bib-0055] Kong, Q. , Ming, W.K. & Mi, X.S. (2021) Refractive outcomes after intravitreal injection of antivascular endothelial growth factor versus laser photocoagulation for retinopathy of prematurity: a meta‐analysis. BMJ Open, 11(2), e042384. 10.1136/bmjopen-2020-042384 PMC7878142 33568373

[aos17541-bib-0056] Kumari, A. , Surve, A. , Azad, S.V. , Chawla, R. , Chandra, P. , Thukral, A. et al. (2021) An observational study of different treatment practices for aggressive posterior retinopathy of prematurity. Journal of Pediatric Ophthalmology and Strabismus, 58(6), 370–376. 34228562 10.3928/01913913-20210423-01

[aos17541-bib-0057] Leng, Y. , Huang, W. , Ren, G. , Cai, C. , Tan, Q. , Liang, Y. et al. (2018) The treatment and risk factors of retinopathy of prematurity in neonatal intensive care units. BMC Ophthalmology, 18(1), 301. 30458733 10.1186/s12886-018-0973-1 PMC6247707

[aos17541-bib-0058] Lepore, D. , Quinn, G.E. , Molle, F. , Baldascino, A. , Orazi, L. , Sammartino, M. et al. (2014) Intravitreal bevacizumab versus laser treatment in type 1 retinopathy of prematurity: report on fluorescein angiographic findings. Ophthalmology, 121(11), 2212–2219. 25001158 10.1016/j.ophtha.2014.05.015

[aos17541-bib-0059] Lepore, D. , Quinn, G.E. , Molle, F. , Orazi, L. , Baldascino, A. , Ji, M.H. et al. (2018) Follow‐up to age 4 years of treatment of type 1 retinopathy of prematurity intravitreal bevacizumab injection versus laser: fluorescein angiographic findings. Ophthalmology, 125(2), 218–226. 28867130 10.1016/j.ophtha.2017.08.005

[aos17541-bib-0060] Li, Z. , Zhang, Y. , Liao, Y. , Zeng, R. , Zeng, P. & Lan, Y. (2018) Comparison of efficacy between anti‐vascular endothelial growth factor (VEGF) and laser treatment in type‐1 and threshold retinopathy of prematurity (ROP). BMC Ophthalmology, 18(1), 19. 29378530 10.1186/s12886-018-0685-6 PMC5789737

[aos17541-bib-0061] Liberati, A. , Altman, D.G. , Tetzlaff, J. , Mulrow, C. , Gøtzsche, P.C. , Ioannidis, J.P.A. et al. (2009) The PRISMA statement for reporting systematic reviews and meta‐analyses of studies that evaluate health care interventions: explanation and elaboration. Annals of Internal Medicine, 151(4), W65–W94. 19622512 10.7326/0003-4819-151-4-200908180-00136

[aos17541-bib-0062] Ling, K.P. , Liao, P.J. , Wang, N.K. , Chao, A.N. , Chen, K.J. , Chen, T.L. et al. (2020) Rates and risk factors for recurrence of retinopathy of prematurity after laser or intravitreal anti‐vascular endothelial growth factor monotherapy. Retina, 40(9), 1793–1803. 31800460 10.1097/IAE.0000000000002663

[aos17541-bib-0063] Linghu, D. , Cheng, Y. , Zhu, X. , Deng, X. , Yin, H. , Jiang, Y. et al. (2022) Comparison of intravitreal anti‐VEGF agents with laser photocoagulation for retinopathy of prematurity of 1,627 eyes in China. Frontiers in Medicine (Lausanne), 9, 911095. 10.3389/fmed.2022.911095 PMC9193577 35712119

[aos17541-bib-0064] Lolas, M. , Tuma, A. , Zanolli, M. , Agurto, R. , Stevenson, R. & Ossandón, D. (2017) Anatomical and refractive outcomes in patients with treated retinopathy of prematurity. Archivos de la Sociedad Española de Oftalmología, 92(10), 472–476. 28624314 10.1016/j.oftal.2016.12.007

[aos17541-bib-0065] Lyu, J. , Zhang, Q. , Chen, C. , Xu, Y. , Ji, X. & Zhao, P. (2019) Ranibizumab injection and laser photocoagulation to treat type 1 retinopathy of prematurity after 40 weeks post menstrual age: a retrospective case series study. BMC Ophthalmology, 19(1), 60. 30808338 10.1186/s12886-019-1067-4 PMC6390561

[aos17541-bib-0066] Mariduena, J. , Ramagopal, M. , Hiatt, M. , Chandra, S. , Laumbach, R. & Hegyi, T. (2022) Vascular endothelial growth factor levels and bronchopulmonary dysplasia in preterm infants. The Journal of Maternal‐Fetal & Neonatal Medicine, 35(8), 1517–1522. 32366142 10.1080/14767058.2020.1760826

[aos17541-bib-0067] Marlow, N. , Reynolds, J.D. , Lepore, D. , Fielder, A.R. , Stahl, A. , Hao, H. et al. (2024) Ranibizumab versus laser therapy for the treatment of very low birthweight infants with retinopathy of prematurity (RAINBOW): five‐year outcomes of a randomised trial. eClinicalMedicine, 71, 102567. 38638400 10.1016/j.eclinm.2024.102567 PMC11024572

[aos17541-bib-0068] Marlow, N. , Stahl, A. , Lepore, D. , Fielder, A. , Reynolds, J.D. , Zhu, Q. et al. (2021) 2‐year outcomes of ranibizumab versus laser therapy for the treatment of very low birthweight infants with retinopathy of prematurity (RAINBOW extension study): prospective follow‐up of an open label, randomised controlled trial. Lancet Child & Adolescent Health, 5(10), 698–707. 34391532 10.1016/S2352-4642(21)00195-4

[aos17541-bib-0069] Mintz‐Hittner, H.A. , Kennedy, K.A. , Chuang, A.Z. & BEAT‐ROP Cooperative Group . (2011) Efficacy of intravitreal bevacizumab for stage 3+ retinopathy of prematurity. The New England Journal of Medicine, 364(7), 603–615. 21323540 10.1056/NEJMoa1007374 PMC3119530

[aos17541-bib-0070] Mori, Y. , Arima, M. , Ueda, E. , Fujiwara, K. , Seki, E. , Nakama, T. et al. (2021) Risk factors for myopia at 1‐year corrected age following laser photocoagulation for retinopathy of prematurity. Eye, 35(10), 2820–2825. 33257802 10.1038/s41433-020-01321-z PMC8452716

[aos17541-bib-0071] Morin, J. , Luu, T.M. , Superstein, R. , Ospina, L.H. , Lefebvre, F. , Simard, M.‐N. et al. (2016) Neurodevelopmental outcomes following bevacizumab injections for retinopathy of prematurity. Pediatrics, 137(4), e20153218. 27244705 10.1542/peds.2015-3218

[aos17541-bib-0072] Morrison, D. , Shaffer, J. , Ying, G.‐S. , Binenbaum, G. & G‐ROP Study Group . (2018) Ocular complications following treatment in the postnatal growth and retinopathy of prematurity (G‐ROP) study. Journal of the American Association for Pediatric Ophthalmology and Strabismus, 22(2), 128–133. 10.1016/j.jaapos.2017.12.005 PMC5915915 29548840

[aos17541-bib-0073] Mueller, B. , Salchow, D.J. , Waffenschmidt, E. , Joussen, A.M. , Schmalisch, G. , Czernik, C. et al. (2017) Treatment of type I ROP with intravitreal bevacizumab or laser photocoagulation according to retinal zone. British Journal of Ophthalmology, 101(3), 365–370. 27301450 10.1136/bjophthalmol-2016-308375

[aos17541-bib-0074] Murakami, T. , Sugiura, Y. , Okamoto, F. , Okamoto, Y. , Kato, A. , Hoshi, S. et al. (2021) Comparison of 5‐year safety and efficacy of laser photocoagulation and intravitreal bevacizumab injection in retinopathy of prematurity. Graefe's Archive for Clinical and Experimental Ophthalmology, 259(9), 2849–2855. 10.1007/s00417-021-05137-9 33744981

[aos17541-bib-0075] Myint, M.Z.Z. , Jia, J. , Adlat, S. , Oo, Z.M. , Htoo, H. , Hayel, F. et al. (2021) Effect of low VEGF on lung development and function. Transgenic Research, 30(1), 35–50. 33394314 10.1007/s11248-020-00223-w

[aos17541-bib-0076] Natarajan, G. , Shankaran, S. , Nolen, T.L. , Sridhar, A. , Kennedy, K.A. , Hintz, S.R. et al. (2019) Neurodevelopmental outcomes of preterm infants with retinopathy of prematurity by treatment. Pediatrics, 144(2), e20183537. 31337693 10.1542/peds.2018-3537 PMC6855825

[aos17541-bib-0077] Ng, Y.S. , Rohan, R. , Sunday, M.E. , Demello, D.E. & D'Amore, P.A. (2001) Differential expression of VEGF isoforms in mouse during development and in the adult. Developmental Dynamics, 220(2), 112–121. 11169844 10.1002/1097-0177(2000)9999:9999<::AID-DVDY1093>3.0.CO;2-D

[aos17541-bib-0078] Nicoară, S.D. , Ștefănuţ, A.C. , Nascutzy, C. , Zaharie, G.C. , Toader, L.E. & Drugan, T.C. (2016) Regression rates following the treatment of aggressive posterior retinopathy of prematurity with bevacizumab versus laser: 8‐year retrospective analysis. Medical Science Monitor, 22, 1192–1209. 27062023 10.12659/MSM.897095 PMC4918525

[aos17541-bib-0079] Nitkin, C.R. , Bamat, N.A. , Lagatta, J. , DeMauro, S.B. , Lee, H.C. , Patel, R.M. et al. (2022) Pulmonary hypertension in preterm infants treated with laser vs anti‐vascular endothelial growth factor therapy for retinopathy of prematurity. JAMA Ophthalmology, 140(11), 1085–1094. 36201183 10.1001/jamaophthalmol.2022.3788 PMC9539731

[aos17541-bib-0080] O'Keeffe, N. , Murphy, J. , Okeeffe, M. & Lanigan, B. (2016) Bevacizumab compared with diode laser in stage 3 posterior retinopathy of prematurity: a 5‐year follow‐up. Irish Medical Journal, 109(2), 355. 27685689

[aos17541-bib-0081] Ortiz‐Seller, A. , Martorell, P. , Barranco, H. , Pascual‐Camps, I. , Morcillo, E. & Ortiz, J.L. (2024) Comparison of different agents and doses of anti‐vascular endothelial growth factors (aflibercept, bevacizumab, conbercept, ranibizumab) versus laser for retinopathy of prematurity: a network meta‐analysis. Survey of Ophthalmology, 69(4), 585–605. 38432359 10.1016/j.survophthal.2024.02.005

[aos17541-bib-0082] Pfeil, J.M. , Barth, T. , Lagrèze, W.A. , Lorenz, B. , Hufendiek, K. , Liegl, R. et al. (2024) Treated cases of retinopathy of prematurity in Germany – 10‐year data from the Retina.net ROP registry. Ophthalmology Retina, 8(6), 579–589. 38104929 10.1016/j.oret.2023.12.002

[aos17541-bib-0083] Pham, B. , Thomas, S.M. , Lillie, E. , Lee, T. , Hamid, J. , Richter, T. et al. (2019) Anti‐vascular endothelial growth factor treatment for retinal conditions: a systematic review and meta‐analysis. BMJ Open, 9(5), e022031. 10.1136/bmjopen-2018-022031 PMC6549720 31142516

[aos17541-bib-0084] Popovic, M.M. , Nichani, P. , Muni, R.H. , Mireskandari, K. , Tehrani, N.N. & Kertes, P.J. (2021) Intravitreal antivascular endothelial growth factor injection versus laser photocoagulation for retinopathy of prematurity: a meta‐analysis of 3,701 eyes. Survey of Ophthalmology, 66(4), 572–584. 33338470 10.1016/j.survophthal.2020.12.002

[aos17541-bib-0085] Raghuram, K. , Isaac, M. , Yang, J. , AlAli, A. , Mireskandari, K. , Ly, L.G. et al. (2019) Neurodevelopmental outcomes in infants treated with intravitreal bevacizumab versus laser. Journal of Perinatology, 39(9), 1300–1308. 31341226 10.1038/s41372-019-0420-z

[aos17541-bib-0086] Reibaldi, M. , Fallico, M. , Avitabile, T. , Bonfiglio, V. , Russo, A. , Castellino, N. et al. (2020) Risk of death associated with intravitreal anti‐vascular endothelial growth factor therapy: a systematic review and meta‐analysis. JAMA Ophthalmology, 138(1), 50–57. 31750861 10.1001/jamaophthalmol.2019.4636 PMC6902107

[aos17541-bib-0087] Rodriguez, S.H. , Peyton, C. , Lewis, K. , Andrews, B. , Greenwald, M.J. , Schreiber, M.D. et al. (2019) Neurodevelopmental outcomes comparing bevacizumab to laser for type 1 ROP. Ophthalmic Surgery, Lasers & Imaging Retina, 50(6), 337–343. 10.3928/23258160-20190605-01 31233150

[aos17541-bib-0088] Roohipoor, R. , Karkhaneh, R. , Riazi‐Esfahani, M. , Dastjani Farahani, A. , Khodabandeh, A. , Ebrahimi Adib, N. et al. (2018) Comparison of intravitreal bevacizumab and laser photocoagulation in the treatment of retinopathy of prematurity. Ophthalmology Retina, 2(9), 942–948. 31047228 10.1016/j.oret.2018.01.017

[aos17541-bib-0089] Roohipoor, R. , Torabi, H. , Karkhaneh, R. & Riazi‐Eafahani, M. (2019) Comparison of intravitreal bevacizumab injection and laser photocoagulation for type 1 zone II retinopathy of prematurity. Journal of Current Ophthalmology, 31(1), 61–65. 30899848 10.1016/j.joco.2018.10.008 PMC6407151

[aos17541-bib-0090] Sankar, M.J. , Sankar, J. & Chandra, P. (2018) Anti‐vascular endothelial growth factor (VEGF) drugs for treatment of retinopathy of prematurity. Cochrane Database of Systematic Reviews, 1(1), CD009734. 29308602 10.1002/14651858.CD009734.pub3 PMC6491066

[aos17541-bib-0091] Sato, T. , Wada, K. , Arahori, H. , Kuno, N. , Imoto, K. , Iwahashi‐Shima, C. et al. (2012) Serum concentrations of bevacizumab (Avastin) and vascular endothelial growth factor in infants with retinopathy of prematurity. American Journal of Ophthalmology, 153(2), 327–333.e1. 21930258 10.1016/j.ajo.2011.07.005

[aos17541-bib-0092] Shah, P.K. , Subramanian, P. , Venkatapathy, N. , Chan, R.V.P. , Chiang, M.F. & Campbell, J.P. (2019) Aggressive posterior retinopathy of prematurity in two cohorts of patients in South India: implications for primary, secondary, and tertiary prevention. Journal of AAPOS, 23(5), 264.e1–264.e4. 10.1016/j.jaapos.2019.05.014 PMC7219568 31521847

[aos17541-bib-0093] Simmons, M. , Wang, J. , Leffler, J.N. , Li, S. , Morale, S.E. , de la Cruz, A. et al. (2021) Longitudinal development of refractive error in children treated with intravitreal bevacizumab or laser for retinopathy of prematurity. Translational Vision Science & Technology, 10(4), 14. 10.1167/tvst.10.4.14 PMC8054622 34003992

[aos17541-bib-0094] Stahl, A. , Lepore, D. , Fielder, A. , Fleck, B. , Reynolds, J.D. , Chiang, M.F. et al. (2019) Ranibizumab versus laser therapy for the treatment of very low birthweight infants with retinopathy of prematurity (RAINBOW): an open‐label randomised controlled trial. Lancet, 394(10208), 1551–1559. 31522845 10.1016/S0140-6736(19)31344-3 PMC12316478

[aos17541-bib-0095] Stahl, A. , Nakanishi, H. , Lepore, D. , Wu, W.C. , Azuma, N. , Jacas, C. et al. (2024) Intravitreal aflibercept vs laser therapy for retinopathy of prematurity. JAMA Network Open, 7(4), e248383. 38687481 10.1001/jamanetworkopen.2024.8383 PMC11061767

[aos17541-bib-0096] Stahl, A. , Sukgen, E.A. , Wu, W.C. , Lepore, D. , Nakanishi, H. , Mazela, J. et al. (2022) Effect of intravitreal aflibercept vs laser photocoagulation on treatment success of retinopathy of prematurity: the FIREFLEYE randomized clinical trial. Journal of the American Medical Association, 328(4), 348–359. 35881122 10.1001/jama.2022.10564 PMC9327573

[aos17541-bib-0097] Sterne, J.A. , Hernán, M.A. , Reeves, B.C. , Savović, J. , Berkman, N.D. , Viswanathan, M. et al. (2016) ROBINS‐I: a tool for assessing risk of bias in non‐randomised studies of interventions. BMJ (Clinical Research Ed.), 355, i4919. 10.1136/bmj.i4919 PMC5062054 27733354

[aos17541-bib-0098] Sterne, J.A.C. , Savović, J. , Page, M.J. , Elbers, R.G. , Blencowe, N.S. , Boutron, I. et al. (2019) RoB 2: a revised tool for assessing risk of bias in randomised trials. BMJ, 366, l4898. 31462531 10.1136/bmj.l4898

[aos17541-bib-0099] Taher, N.O. , Ghaddaf, A.A. , Al‐Ghamdi, S.A. , Homsi, J.J. , Al‐Harbi, B.J. , Alomari, L.K. et al. (2022) Intravitreal anti‐vascular endothelial growth factor injection for retinopathy of prematurity: a systematic review and meta‐analysis. Frontiers in Medicine (Lausanne), 9, 884608. 10.3389/fmed.2022.884608 PMC9124790 35615084

[aos17541-bib-0100] Tan, Q.Q. , Christiansen, S.P. & Wang, J. (2019) Development of refractive error in children treated for retinopathy of prematurity with anti‐vascular endothelial growth factor (anti‐VEGF) agents: a meta‐analysis and systematic review. PLoS One, 14(12), e0225643. 31790445 10.1371/journal.pone.0225643 PMC6886775

[aos17541-bib-0101] Thulliez, M. , Angoulvant, D. , Le Lez, M.L. , Jonville‐Bera, A.P. , Pisella, P.J. , Gueyffier, F. et al. (2014) Cardiovascular events and bleeding risk associated with intravitreal antivascular endothelial growth factor monoclonal antibodies: systematic review and meta‐analysis. JAMA Ophthalmology, 132(11), 1317–1326. 25058694 10.1001/jamaophthalmol.2014.2333

[aos17541-bib-0102] Tomioka, M. , Murakami, T. , Okamoto, F. , Kinoshita, T. , Shinomiya, K. , Nishi, T. et al. (2024) Five‐year visual outcome of treatment for retinopathy of prematurity in infants weighing <500 g at birth: a multicenter cohort study from J‐CREST. Retina, 44(4), 652–658. 38064668 10.1097/IAE.0000000000004016

[aos17541-bib-0103] Tsai, C.Y. , Yeh, P.T. , Tsao, P.N. , Chung, Y.C.E. , Chang, Y.S. & Lai, T.T. (2021) Neurodevelopmental outcomes after bevacizumab treatment for retinopathy of prematurity: a meta‐analysis. Ophthalmology, 128(6), 877–888. 33212122 10.1016/j.ophtha.2020.11.012

[aos17541-bib-0104] Ueta, T. , Noda, Y. , Toyama, T. , Yamaguchi, T. & Amano, S. (2014) Systemic vascular safety of ranibizumab for age‐related macular degeneration: systematic review and meta‐analysis of randomized trials. Ophthalmology, 121(11), 2193–2203.e1–7. 25023760 10.1016/j.ophtha.2014.05.022

[aos17541-bib-0105] Vujanovic, M.S. , Stankovic‐Babic, G.L. , Oros, A. , Zlatanovic, G.D. , Jovanovic, P. , Cekic, S.P. et al. (2017) Refractive errors in premature infants with retinopathy of prematurity after anti‐vascular endothelial growth factor (anti‐VEGF) therapy. Vojnosanitetski Pregled, 74(4), 323–328.

[aos17541-bib-0106] Walz, J.M. , Bemme, S. , Pielen, A. , Aisenbrey, S. , Breuß, H. , Alex, A.F. et al. (2016) The German ROP registry: data from 90 infants treated for retinopathy of prematurity. Acta Ophthalmologica, 94(8), e744–e752. 27197876 10.1111/aos.13069

[aos17541-bib-0107] Walz, J.M. , Bemme, S. , Reichl, S. , Akman, S. , Breuß, H. , Süsskind, D. et al. (2018) Behandelte Frühgeborenenretinopathie in Deutschland. Der Ophthalmologe, 115(6), 476–488. 29637302 10.1007/s00347-018-0701-5

[aos17541-bib-0108] Wang, S. , Liu, J. , Zhang, X. , Liu, Y. , Li, J. , Wang, H. et al. (2024) Global, regional and national burden of retinopathy of prematurity among childhood and adolescent: a spatiotemporal analysis based on the global burden of disease study 2019. BMJ Paediatrics Open, 8(1), e002267. 38184302 10.1136/bmjpo-2023-002267 PMC10773439

[aos17541-bib-0109] Wang, S.D. , Zhang, G.M. & Shenzhen Screening for Retinopathy of Prematurity Cooperative Group . (2020) Laser therapy versus intravitreal injection of anti‐VEGF agents in monotherapy of ROP: a meta‐analysis. International Journal of Ophthalmology, 13(5), 806–815. 32420230 10.18240/ijo.2020.05.17 PMC7201341

[aos17541-bib-0110] Wardati, H. , Khadijah, M. , Nurul‐Farhana, M. , Karimmah, W. , Lai, Y.K.I. , Syahmi, M.R. et al. (2024) Comparison of intravitreal ranibizumab and laser photocoagulation in the treatment of type I retinopathy of prematurity in Malaysia: a one‐year follow‐up study. Cureus, 16(7), e63712. 38966779 10.7759/cureus.63712 PMC11223661

[aos17541-bib-0111] Winter, K. , Pfeil, J.M. , Engmann, H. , Aisenbrey, S. , Lorenz, B. , Hufendiek, K. et al. (2024) Comparability of input parameters in the German retina.Net ROP registry and the EU‐ROP registry – an exemplary comparison between 2011 and 2021. Acta Ophthalmologica, 102(3), e314–e321. 37725047 10.1111/aos.15753

[aos17541-bib-0112] Xu, Y. , Deng, G. , Zhang, J. , Zhu, J. , Liu, Z. , Xu, F. et al. (2023) Efficacy of four anti‐vascular endothelial growth factor agents and laser treatment for retinopathy of prematurity: a network meta‐analysis. Biomolecules & Biomedicine, 24(4), 676–687. 37976345 10.17305/bb.2023.9829 PMC11293245

[aos17541-bib-0113] Yenice, E.K. , Petriçli, İ.S. & Kara, C. (2023) One‐year refractive outcomes after intravitreal bevacizumab versus laser photocoagulation for retinopathy of prematurity. International Ophthalmology, 43(7), 2197–2202. 36522564 10.1007/s10792-022-02615-9

[aos17541-bib-0114] Zayek, M. , Parker, K. , Rydzewska, M. , Rifai, A. , Bhat, R. & Eyal, F. (2020) Bevacizumab for retinopathy of prematurity: 2‐year neurodevelopmental follow‐up. American Journal of Perinatology, 38(11), 1158–1166. 32446264 10.1055/s-0040-1710556

[aos17541-bib-0115] Zhang, G. , Yang, M. , Zeng, J. , Vakros, G. , Su, K. , Chen, M. et al. (2017) Comparison of intravitreal injection of ranibizumab versus laser therapy for zone II treatment‐requiring retinopathy of prematurity. Retina, 37(4), 710–717. 27529839 10.1097/IAE.0000000000001241 PMC5388026

[aos17541-bib-0116] Zhang, M.H. , Blair, M.P. , Ham, S.A. & Rodriguez, S. (2020) Two‐year outcomes comparing anti‐VEGF injections to laser for ROP using a commercial claims database. Ophthalmic Surgery, Lasers & Imaging Retina, 51, 486–493. 10.3928/23258160-20200831-02 32955587

