# Supplementary material for: Impact of anti‐VEGF therapy versus laser therapy on mortality and treatment outcomes in retinopathy of prematurity: A systematic review and meta‐analysis
Source: Acta Ophthalmol. 2025 Jun 28;104(1):e1–e17. doi: 10.1111/aos.17541 (PMC12803688; doi:10.1111/aos.17541)

**Impact of Anti-VEGF Therapy versus Laser Therapy on Mortality and Treatment Outcomes in Retinopathy of Prematurity**

A Systematic Review and Meta-Analysis

**Supplementary Table 1**. Search strategies

**Supplementary Table 2**. Quality assessment of randomized controlled trials

**Supplementary Table 3**. Quality assessment of observational studies

**Supplementary Figure 1.** Funnel plot for the mortality rate

**Supplementary Figure 2.** The cause of death and the relative proportions in the anti-VEGF and laser groups

**Supplementary Figure 3.** Forest plot of retina dragging/ macular dragging/ macular traction comparing anti-VEGF and laser groups

**Supplementary Figure 4.** Forest plot of the incidence of retinal holes comparing anti-VEGF and laser groups

**Supplementary Figure 5.** Forest plot of the incidence of macular fold/ retinal fold comparing anti-VEGF and laser groups

**Supplementary Figure 6.** Forest plot of the incidence of macular ectopia comparing anti-VEGF and laser groups

**Supplementary Figure 7.** Forest plot of the incidence of hemorrhage comparing anti-VEGF and laser groups

**Supplementary Figure 8.** Forest plot of the incidence of uveitis comparing anti-VEGF and laser groups

**Supplementary Figure 9.** Forest plot of the incidence of endophthalmitis comparing anti-VEGF and laser groups

**Supplementary Figure 10.** Forest plot of the incidence of cataract/ lens opacity comparing anti-VEGF and laser groups

**Supplementary Figure 11.** Forest plot of the incidence of keratitis comparing anti-VEGF and laser groups

**Supplementary Figure 12.** Forest plot of the incidence of corneal erosion comparing anti-VEGF and laser groups

**Supplementary Figure 13.** Forest plot of the incidence of corneal opacity comparing anti-VEGF and laser groups

**Supplementary Table 1**. Search strategies

| **Database** | **#** | **Search syntax** | **Citations found** |
| --- | --- | --- | --- |
| 1. Embase | 1 | (prematurity NEAR/2 retinopath*):ti,ab,de,kw | 11,827 |
|  | 2 | 'retrolental fibroplasia':ti,ab,de,kw | 14,394 |
|  | 3 | 'retrolental dysplasia':ti,ab,de,kw | 0 |
|  | 4 | 'retrolental fibrosis':ti,ab,de,kw | 5 |
|  | 5 | rop:ti,ab,de,kw | 9,644 |
|  | 6 | 'retrolental fibroplasia'/exp | 14,394 |
|  | 7 | #1 OR #2 OR #3 OR #4 OR #5 OR #6 | 19,016 |
|  | 8 | vegf*:ti,ab,de,kw | 151,410 |
|  | 9 | (anti NEAR/2 angiogen*):ti,ab,de,kw | 26,678 |
|  | 10 | (endothelial NEAR/3 growth NEAR/3 factor*):ti,ab,de,kw | 103,994 |
|  | 11 | aflibercept:ti,ab,de,kw | 10,726 |
|  | 12 | bevacizumab:ti,ab,de,kw | 83,793 |
|  | 13 | brolucizumab:ti,ab,de,kw | 844 |
|  | 14 | conbercept:ti,ab,de,kw | 773 |
|  | 15 | faricimab:ti,ab,de,kw | 617 |
|  | 16 | pegaptanib:ti,ab,de,kw | 2,648 |
|  | 17 | ranibizumab:ti,ab,de,kw | 14,107 |
|  | 18 | 'vasculotropin antibody'/exp | 7,082 |
|  | 19 | 'vasculotropin inhibitor'/exp | 321,657 |
|  | 20 | 'angiogenesis inhibitor'/exp | 600,184 |
|  | 21 | #8 OR #9 OR #10 OR #11 OR #12 OR #13 OR #14 OR #15 OR #16 OR #17 OR #18 OR #19 OR #20 | 741,003 |
|  | 22 | laser*:ti,ab,de,kw | 499,556 |
|  | 23 | photocoagulat*:ti,ab,de,kw | 15,509 |
|  | 24 | coagulat*:ti,ab,de,kw OR argon:ti,ab,de,kw OR diode:ti,ab,de,kw | 287,263 |
|  | 25 | 'laser coagulation'/exp | 27,379 |
|  | 26 | 'laser'/exp | 520,490 |
|  | 27 | 'ophthalmic laser'/exp | 4,962 |
|  | 28 | 'ophthalmic argon laser'/exp | 234 |
|  | 29 | 'ophthalmic diode laser'/exp | 263 |
|  | 30 | #22 OR #23 OR #24 OR #25 OR #26 OR #27 OR #28 OR #29 | 753,195 |
|  | 31 | #7 AND #21 | 3,106 |
|  | 32 | #7 AND #21 AND #30 | 1,362 |
| 2) MEDLINE | 1 | (prematurity adj2 retinopath*).mp. | 10,671 |
|  | 2 | "retrolental fibroplasia".mp. | 1,058 |
|  | 3 | "retrolental dysplasia".mp. | 0 |
|  | 4 | "retrolental fibrosis".mp. | 4 |
|  | 5 | ROP.mp. | 7,464 |
|  | 6 | exp "retinopathy of prematurity"/ | 7,433 |
|  | 7 | 1 or 2 or 3 or 4 or 5 or 6 | 13,275 |
|  | 8 | VEGF*.mp. | 100,715 |
|  | 9 | (anti adj2 angiogen*).mp. | 17,376 |
|  | 10 | (endothelial adj3 growth adj3 factor*).mp. | 105,901 |
|  | 11 | aflibercept.mp. | 3,866 |
|  | 12 | bevacizumab.mp. | 24,914 |
|  | 13 | brolucizumab.mp. | 437 |
|  | 14 | conbercept.mp. | 367 |
|  | 15 | faricimab.mp. | 259 |
|  | 16 | pegaptanib.mp. | 680 |
|  | 17 | ranibizumab.mp. | 6,905 |
|  | 18 | exp "angiogenesis Inhibitors"/ | 71,263 |
|  | 19 | exp "angiogenesis Inducing Agents"/ | 7,406 |
|  | 20 | exp "endothelial Growth Factors"/ | 8,339 |
|  | 21 | exp "vascular Endothelial Growth Factors"/ | 67,785 |
|  | 22 | 8 or 9 or 10 or 11 or 12 or 13 or 14 or 15 or 16 or 17 or 18 or 19 or 20 or 21 | 200,766 |
|  | 23 | laser*.mp. | 387,207 |
|  | 24 | photocoagulat*.mp. | 11,138 |
|  | 25 | (coagulat* or argon or diode).mp. | 242,011 |
|  | 26 | exp "lasers"/ | 62,442 |
|  | 27 | exp "laser coagulation"/ | 8,425 |
|  | 28 | exp "laser Therapy"/ | 69,209 |
|  | 29 | 23 or 24 or 25 or 26 or 27 or 28 | 597,065 |
|  | 30 | 7 and 22 | 1,777 |
|  | 31 | 7 and 22 and 29 | 704 |
| 3) CENTRAL | 1 | (prematurity near/1 retinopath*):ti,ab,kw | 33 |
|  | 2 | ("retrolental fibroplasia"):ti,ab,kw | 547 |
|  | 3 | ("retrolental dysplasia"):ti,ab,kw | 0 |
|  | 4 | ("retrolental fibrosis"):ti,ab,kw | 0 |
|  | 5 | ROP:ti,ab,kw | 952 |
|  | 6 | [mh "retinopathy of prematurity"] | 551 |
|  | 7 | #1 OR #2 OR #3 OR #4 OR #5 OR #6 | 1,417 |
|  | 8 | (VEGF*):ti,ab,kw | 7,257 |
|  | 9 | (anti near/1 angiogen*):ti,ab,kw | 879 |
|  | 10 | (endothelial near/2 growth near/2 factor*):ti,ab,kw | 5,722 |
|  | 11 | (aflibercept):ti,ab,kw | 1,281 |
|  | 12 | (bevacizumab):ti,ab,kw | 8,180 |
|  | 13 | (brolucizumab):ti,ab,kw | 108 |
|  | 14 | (conbercept):ti,ab,kw | 179 |
|  | 15 | (faricimab):ti,ab,kw | 102 |
|  | 16 | (pegaptanib):ti,ab,kw | 165 |
|  | 17 | (ranibizumab):ti,ab,kw | 2,412 |
|  | 18 | [mh "angiogenesis Inhibitors"] | 2,139 |
|  | 19 | [mh "angiogenesis Inducing Agents"] | 70 |
|  | 20 | [mh "endothelial growth factors"] | 226 |
|  | 21 | [mh "vascular endothelial growth factors"] | 2,306 |
|  | 22 | #8 OR #9 OR #10 OR #11 OR #12 OR #13 OR #14 OR #15 OR #16 OR #17 OR #18 OR #19 OR #20 OR #21 | 18,035 |
|  | 23 | (laser*):ti,ab,kw | 26,337 |
|  | 24 | (photocoagulat*):ti,ab,kw | 1,853 |
|  | 25 | (coagulat* or argon or diode):ti,ab,kw | 18,721 |
|  | 26 | [mh "lasers"] | 3,898 |
|  | 27 | [mh "laser coagulation"] | 759 |
|  | 28 | [mh "laser therapy"] | 6,179 |
|  | 29 | #23 OR #24 OR #25 OR #26 OR #27 OR #28 | 40,988 |
|  | 30 | #7 AND #22 | 151 |
|  | 31 | #7 AND #22 AND #29 | 112 |
| 4) Scopus | 1 | TITLE-ABS-KEY ( prematurity W/1 retinopath* ) | 12,415 |
|  | 2 | TITLE-ABS-KEY ( "retrolental fibroplasia" ) | 12,079 |
|  | 3 | TITLE-ABS-KEY ( "retrolental dysplasia" ) | 0 |
|  | 4 | TITLE-ABS-KEY ( "retrolental fibrosis" ) | 8 |
|  | 5 | TITLE-ABS-KEY ( rop ) | 16,555 |
|  | 6 | #1 OR #2 OR #3 OR #4 OR #5 | 26,045 |
|  | 7 | TITLE-ABS-KEY ( vegf* ) | 116,080 |
|  | 8 | TITLE-ABS-KEY ( anti AND near/1 AND angiogen* ) | 240 |
|  | 9 | TITLE-ABS-KEY ( endothelial AND near/2 AND growth AND near/2 AND factor* ) | 650 |
|  | 10 | TITLE-ABS-KEY ( aflibercept ) | 8,681 |
|  | 11 | TITLE-ABS-KEY ( bevacizumab ) | 66,858 |
|  | 12 | TITLE-ABS-KEY ( brolucizumab ) | 752 |
|  | 13 | TITLE-ABS-KEY ( conbercept ) | 835 |
|  | 14 | TITLE-ABS-KEY ( faricimab ) | 453 |
|  | 15 | TITLE-ABS-KEY ( pegaptanib ) | 2,598 |
|  | 16 | TITLE-ABS-KEY ( ranibizumab ) | 12,425 |
|  | 17 | #7 OR #8 OR #9 OR #10 OR #11 OR #12 OR #13 OR #14 OR #15 OR #16 | 177,803 |
|  | 18 | TITLE-ABS-KEY ( laser* ) | 1,678,982 |
|  | 19 | TITLE-ABS-KEY ( photocoagulat* ) | 15,364 |
|  | 20 | TITLE-ABS-KEY ( coagulat* OR argon OR diode ) | 818,168 |
|  | 21 | #18 OR #19 OR #20 | 2,331,171 |
|  | 22 | #6 AND #17 | 2,137 |
|  | 23 | #6 AND #17 AND #21 | 1,023 |

**Supplementary Table 2**. Quality assessment of randomized controlled trials

**2a.** Summary of risk of bias


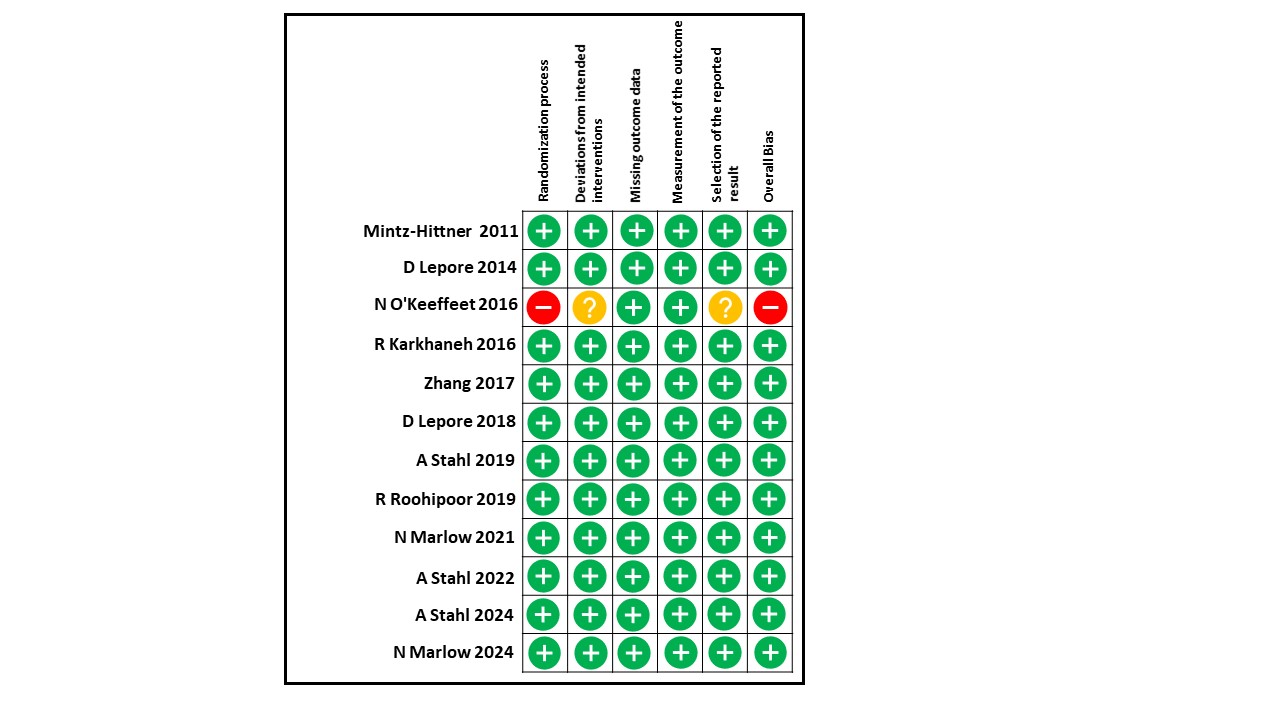


**2b.** Graph of risk of bias


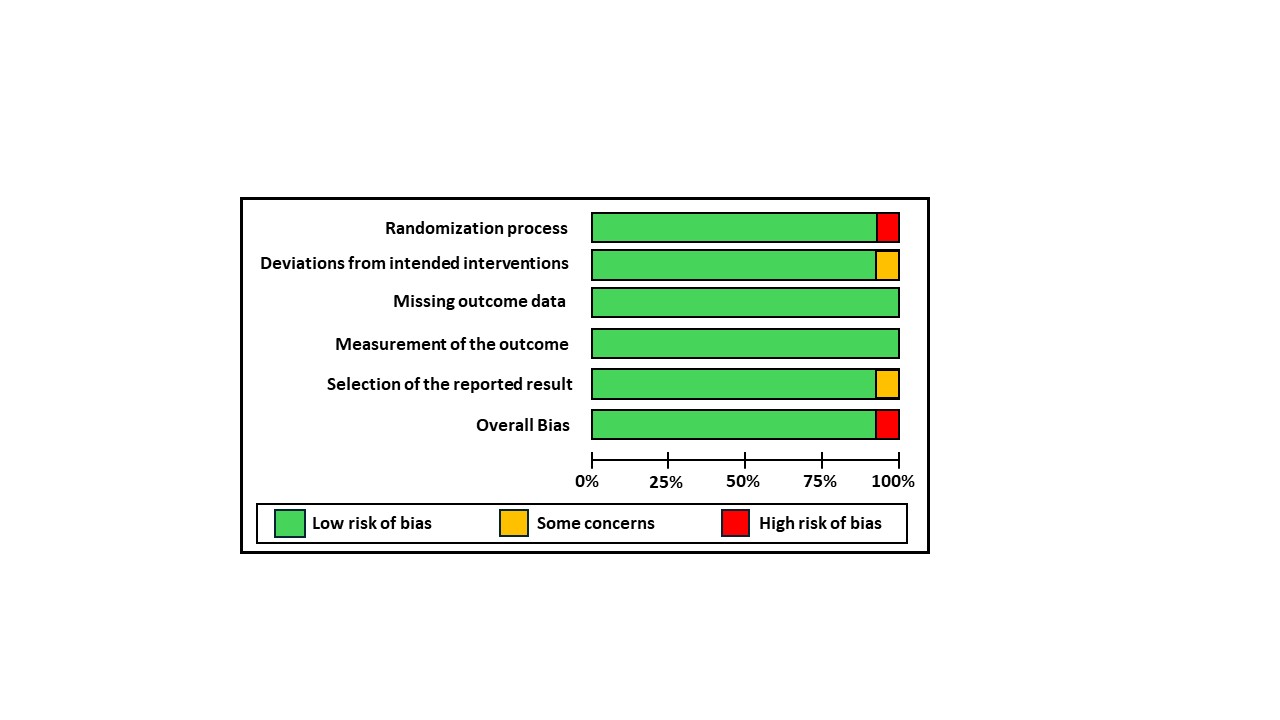


**Supplementary Table 3**. Quality assessment of observational studies

|  | **Study** | **Year** | **Pre-intervention and at-intervention domains** | | | **Post-intervention domains** | | | | **Overall risk of bias** |
| --- | --- | --- | --- | --- | --- | --- | --- | --- | --- | --- |
|  |  |  | Bias due to confounding | Bias in selection of participants in the study | Bias in classification of interventions | Bias due to deviations from intended interventions | Bias due to missing data | Bias in measurement of outcomes | Bias in selection of the reported result |  |
| 1 | Harder | 2013 | M | L | L | L | L | L | L | M |
| 2 | Geloneck | 2014 | M | L | L | L | L | L | L | M |
| 3 | Hwang | 2015 | M | L | L | L | L | L | L | M |
| 4 | Kong (2) | 2015 | M | L | L | L | L | L | L | M |
| 5 | Gunay | 2015 | M | L | L | L | L | L | L | M |
| 6 | Isaac | 2015 | M | L | L | L | M | L | L | M |
| 7 | Chan | 2016 | S | L | L | L | L | M | L | S |
| 8 | Morin | 2016 | M | L | L | M | M | L | L | M |
| 9 | Walz | 2016 | M | M | L | L | M | M | L | M |
| 10 | Gunay (1) | 2016 | M | L | L | L | L | L | L | M |
| 11 | Gunay (2) | 2016 | M | L | L | L | L | L | L | M |
| 12 | Nicoara | 2016 | M | M | L | L | L | L | M | M |
| 13 | Kabatas | 2017 | M | L | L | L | L | L | L | M |
| 14 | Mueller | 2017 | M | L | L | L | L | L | L | M |
| 15 | Gunay | 2017 | M | L | L | L | L | L | L | M |
| 16 | Lolas | 2017 | M | L | L | L | L | L | L | M |
| 17 | Vujanović | 2017 | M | L | L | L | L | L | L | M |
| 18 | Morrison | 2018 | M | L | L | L | L | M | L | M |
| 19 | Adams | 2018 | M | L | M | L | M | L | L | M |
| 20 | Kang | 2018 | M | L | L | L | L | M | L | M |
| 21 | Walz | 2018 | M | M | L | L | M | M | L | M |
| 22 | Kennedy | 2018 | M | L | L | L | L | L | L | M |
| 23 | Blair | 2018 | M | L | L | L | L | L | L | M |
| 24 | Arfat | 2018 | M | M | L | L | L | L | L | M |
| 25 | Roohipoor | 2018 | M | L | L | L | M | L | L | M |
| 26 | Leng | 2018 | M | L | L | L | L | L | L | M |
| 27 | Natarajan | 2019 | M | L | L | L | L | L | L | M |
| 28 | Barry | 2019 | M | L | L | L | L | L | L | M |
| 29 | Kang | 2019 | M | L | L | L | L | L | L | M |
| 30 | Raghuram | 2019 | M | L | L | L | L | L | L | M |
| 31 | Lyu | 2019 | M | L | L | L | L | L | L | M |
| 32 | Shah | 2019 | M | L | L | L | L | L | L | M |
| 33 | Rodriguez | 2019 | M | L | L | L | M | L | L | M |
| 34 | Demir | 2019 | M | L | L | M | L | L | L | M |
| 35 | Ekinci | 2020 | M | L | L | L | L | L | L | M |
| 36 | Barry | 2020 | M | L | L | M | L | L | L | M |
| 37 | Ling | 2020 | M | L | L | L | L | L | L | M |
| 38 | Zayek | 2020 | M | L | L | L | M | L | L | M |
| 39 | Zhang | 2020 | M | L | L | M | M | L | L | M |
| 40 | Chmielarz-Czarnocińska | 2021 | M | L | L | S | M | L | L | S |
| 41 | Kumari | 2021 | M | L | L | L | L | L | L | M |
| 42 | Barry | 2021 | M | L | L | L | L | L | L | M |
| 43 | Simmons | 2021 | M | L | L | L | L | L | L | M |
| 44 | Murakami | 2021 | M | L | L | L | L | L | L | M |
| 45 | Demir | 2021 | M | L | L | L | L | L | L | M |
| 46 | Mori | 2021 | M | L | L | M | M | L | L | M |
| 47 | Elabbasy | 2022 | M | L | L | L | M | L | L | M |
| 48 | Gundlach | 2022 | M | L | L | M | M | L | L | M |
| 49 | Nitkin | 2022 | M | L | L | L | L | L | L | M |
| 50 | Linghu | 2022 | M | L | L | L | L | L | L | M |
| 51 | Chou | 2022 | M | L | L | L | M | L | L | M |
| 52 | Ahn | 2022 | M | L | L | M | M | L | L | M |
| 53 | Celik | 2022 | M | L | L | L | L | L | L | M |
| 54 | Yenice | 2023 | M | L | L | L | L | L | L | M |
| 55 | Pfeil | 2024 | M | L | L | L | L | L | L | M |
| 56 | Winter | 2024 | M | L | L | L | L | L | L | M |
| 57 | Tomioka | 2024 | M | M | L | L | L | L | L | M |
| 58 | Wardati | 2024 | M | L | L | L | L | L | L | M |
| L: low risk of bias; M: moderate risk of bias; S: serious risk of bias | | | | | | | | | | |

**Supplementary Figure 1.** Funnel plot for the mortality rate


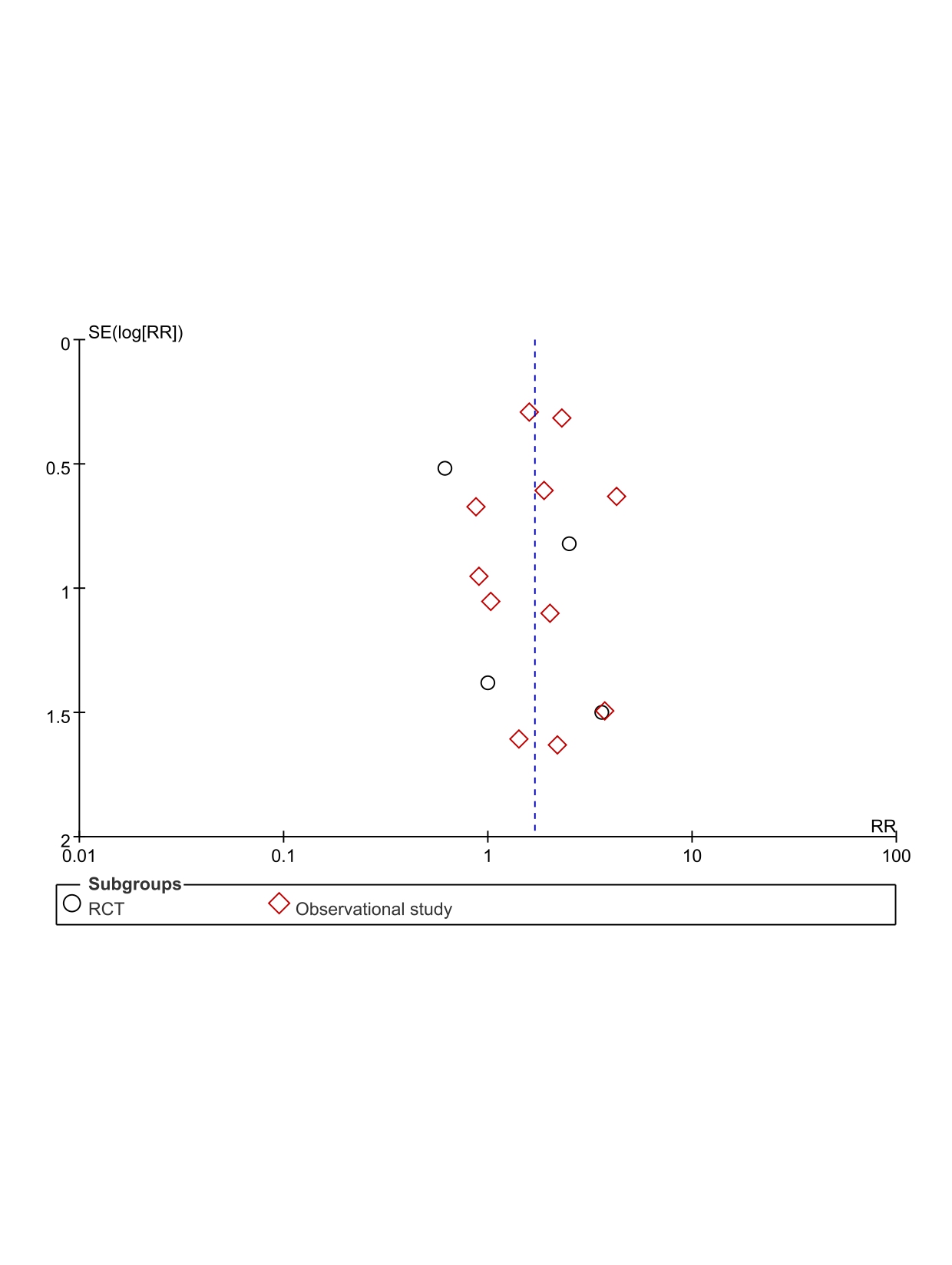


**Supplementary Figure 2.** The causes of death and the relative proportions in the anti-VEGF and laser groups

**2a.** The causes of death in the anti-VEGF group

| **Intervention** | **System** | **Cause of death** | **Number** |
| --- | --- | --- | --- |
| anti-VEGF | Respiratory | Bronchopulmonary dysplasia | 18 |
| anti-VEGF | Respiratory | Low oxygen | 2 |
| anti-VEGF | Respiratory | Respiratory failure | 9 |
| anti-VEGF | Respiratory | Aspiration | 1 |
| anti-VEGF | Respiratory | Bronchopneumonia | 1 |
| anti-VEGF | Respiratory | Bronchiolitis | 1 |
| anti-VEGF | Respiratory | Pulmonary complications | 1 |
| anti-VEGF | Cardiovascular | Cardiac arrest | 1 |
| anti-VEGF | Nervous | Intraventricular hemorrhage | 4 |
| anti-VEGF | Nervous | Periventricular leucomalacia | 1 |
| anti-VEGF | Digestive | Short bowel syndrome | 2 |
| anti-VEGF | Digestive | Necrotizing enterocolitis | 1 |
| anti-VEGF | Renal | Renal failure | 1 |
| anti-VEGF | Systemic | Sepsis | 2 |
| anti-VEGF | Multi-system | Multi-system complications | 2 |
| anti-VEGF | No report | No report | 59 |

**2b.** The proportions of the causes of death in the anti-VEGF group

**2c.** The causes of death in the laser group

| **Intervention** | **System** | **Cause of death** | **Number** |
| --- | --- | --- | --- |
| Laser | Respiratory | Bronchopulmonary dysplasia | 3 |
| Laser | Respiratory | Pulmonary vein stenosis | 1 |
| Laser | Respiratory | Respiratory failure | 3 |
| Laser | Respiratory | Pulmonary complications | 1 |
| Laser | Cardiovascular | Cardiac arrest | 2 |
| Laser | Cardiovascular | Acute decompensated heart failure | 1 |
| Laser | Digestive | Hepatic failure | 1 |
| Laser | Digestive | Necrotizing enterocolitis | 1 |
| Laser | Systemic | Sepsis | 3 |
| Laser | Multi-system | Multi-system complications | 1 |
| Laser | No report | No report | 49 |

**2d.** The proportions of the causes of death in the laser group

**Supplementary Figure 3.** Forest plot of retina dragging, macular dragging, or macular traction comparing anti-VEGF and laser groups. CI= confidence interval


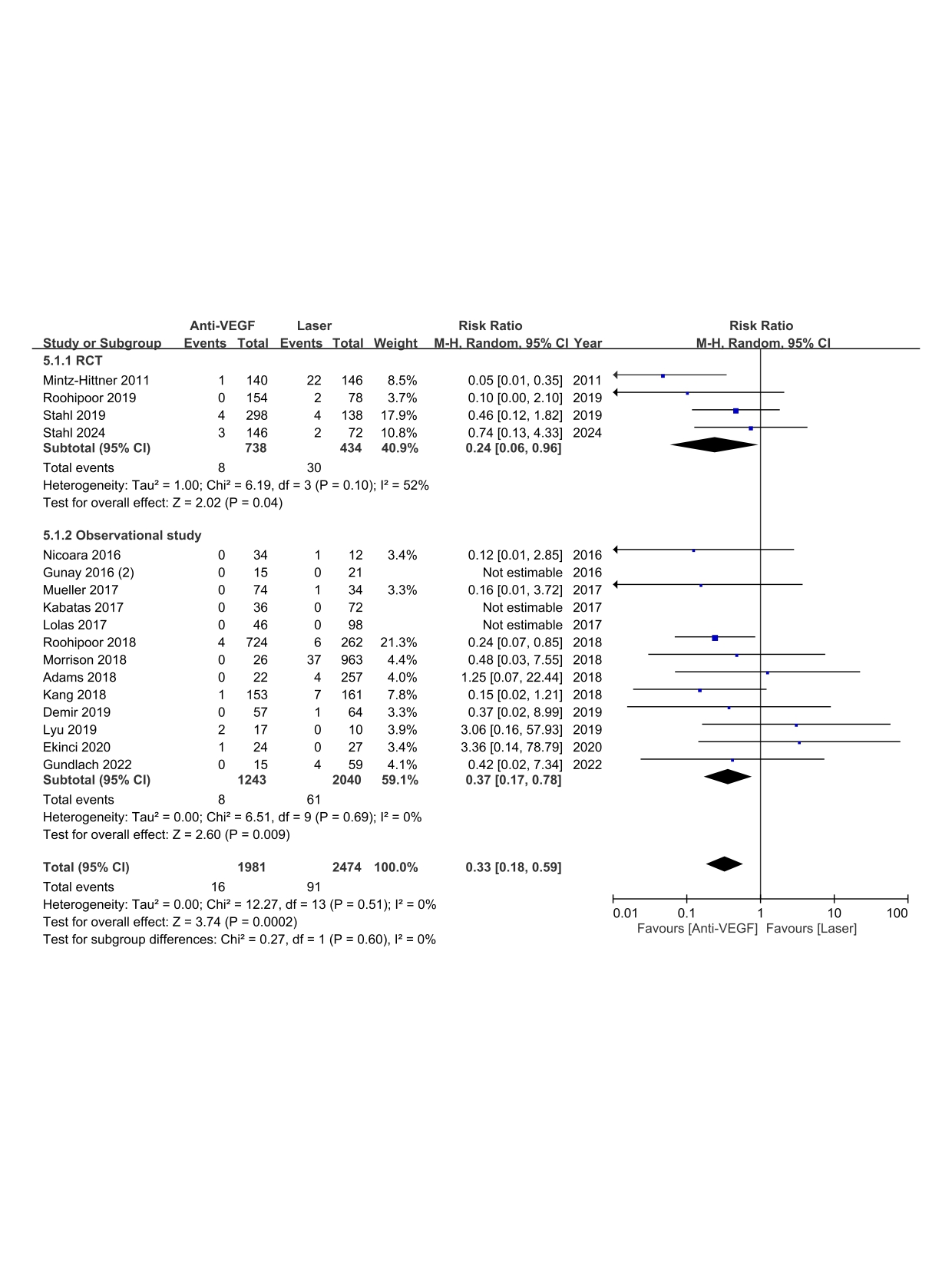


**Supplementary Figure 4.** Forest plot of the incidence of retinal holes comparing anti-VEGF and laser groups. CI= confidence interval


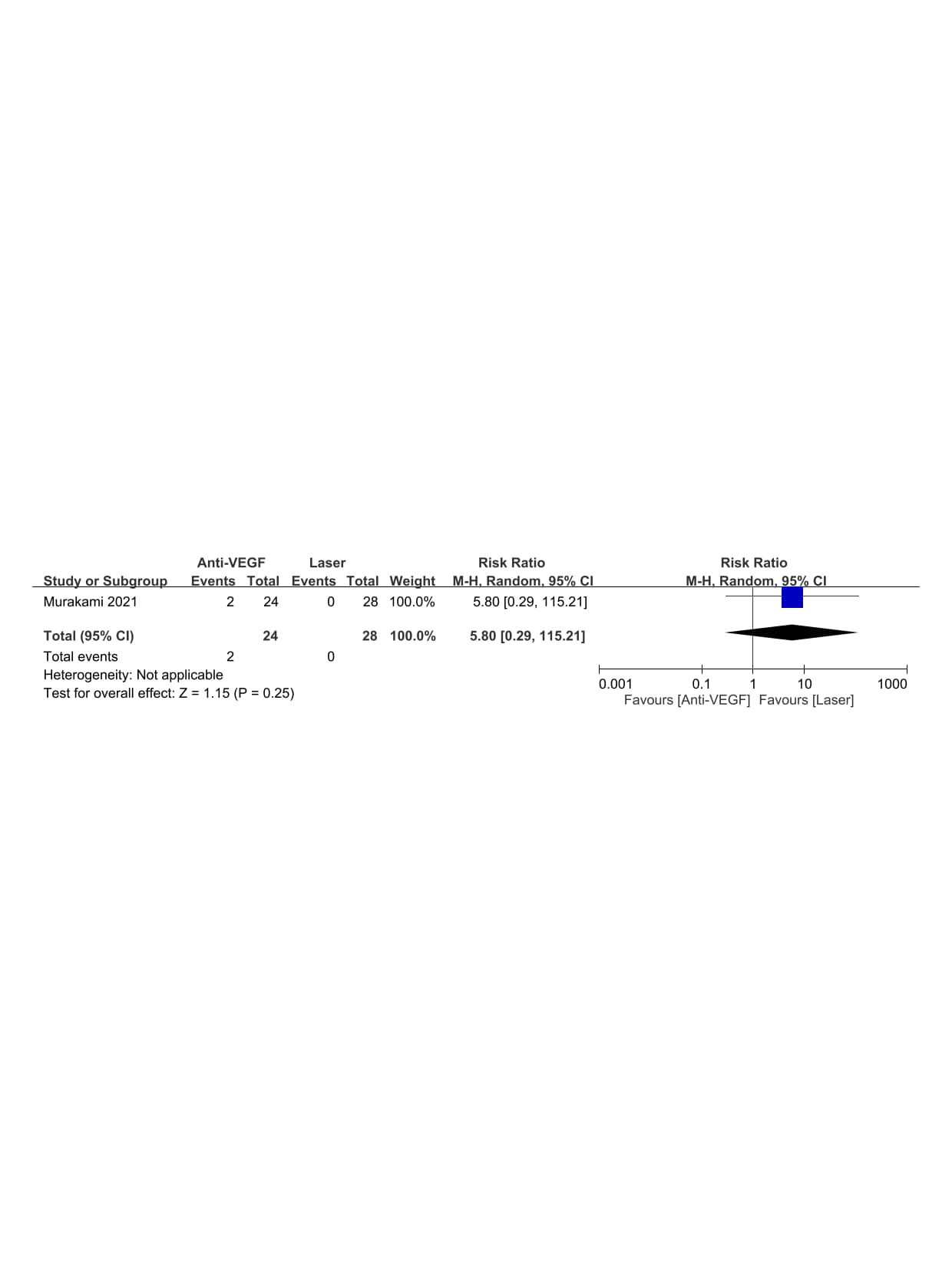


**Supplementary Figure 5.** Forest plot of the incidence of macular fold or retinal fold comparing anti-VEGF and laser groups. CI= confidence interval


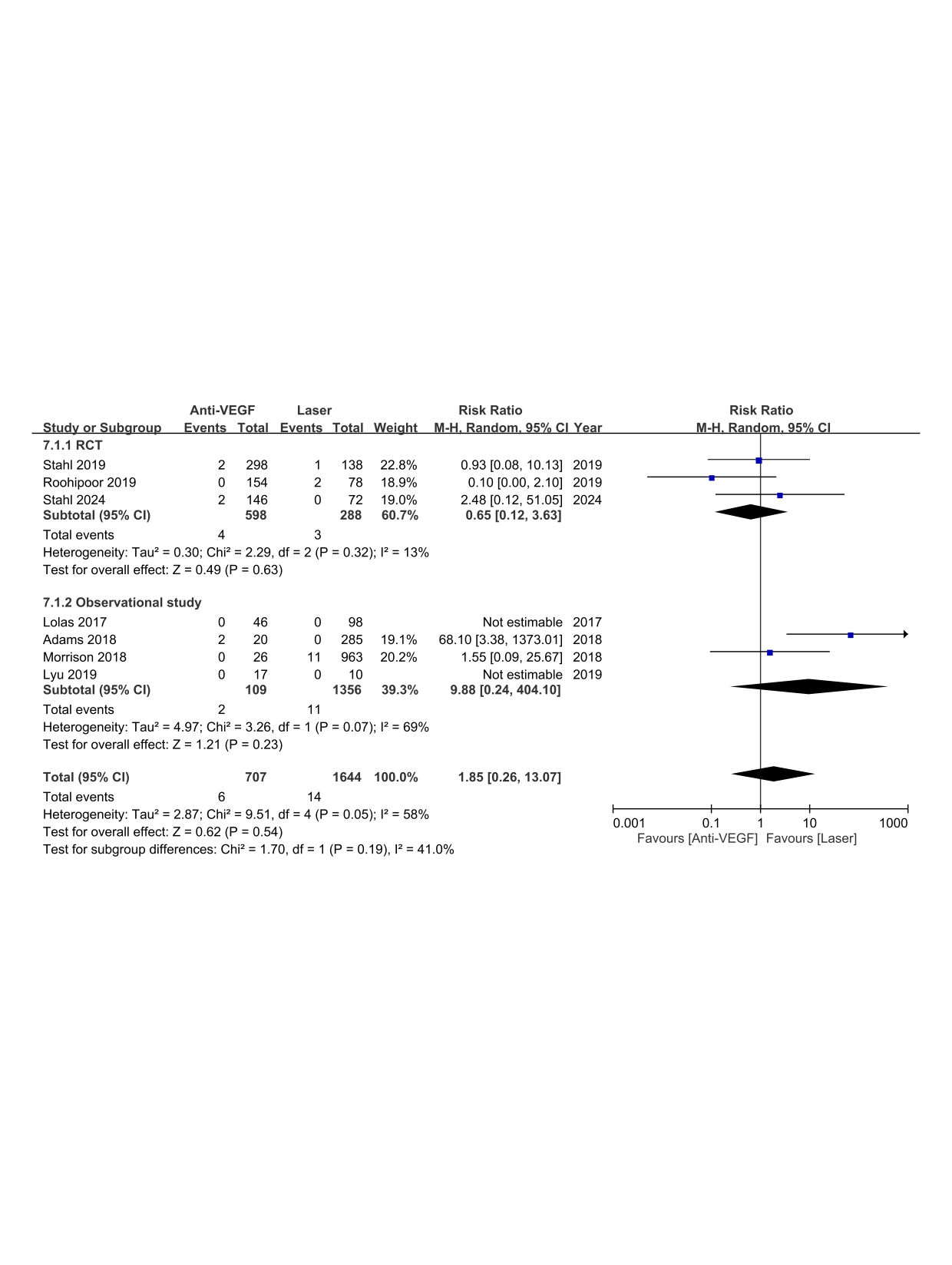


**Supplementary Figure 6.** Forest plot of the incidence of macular ectopia comparing anti-VEGF and laser groups. CI= confidence interval


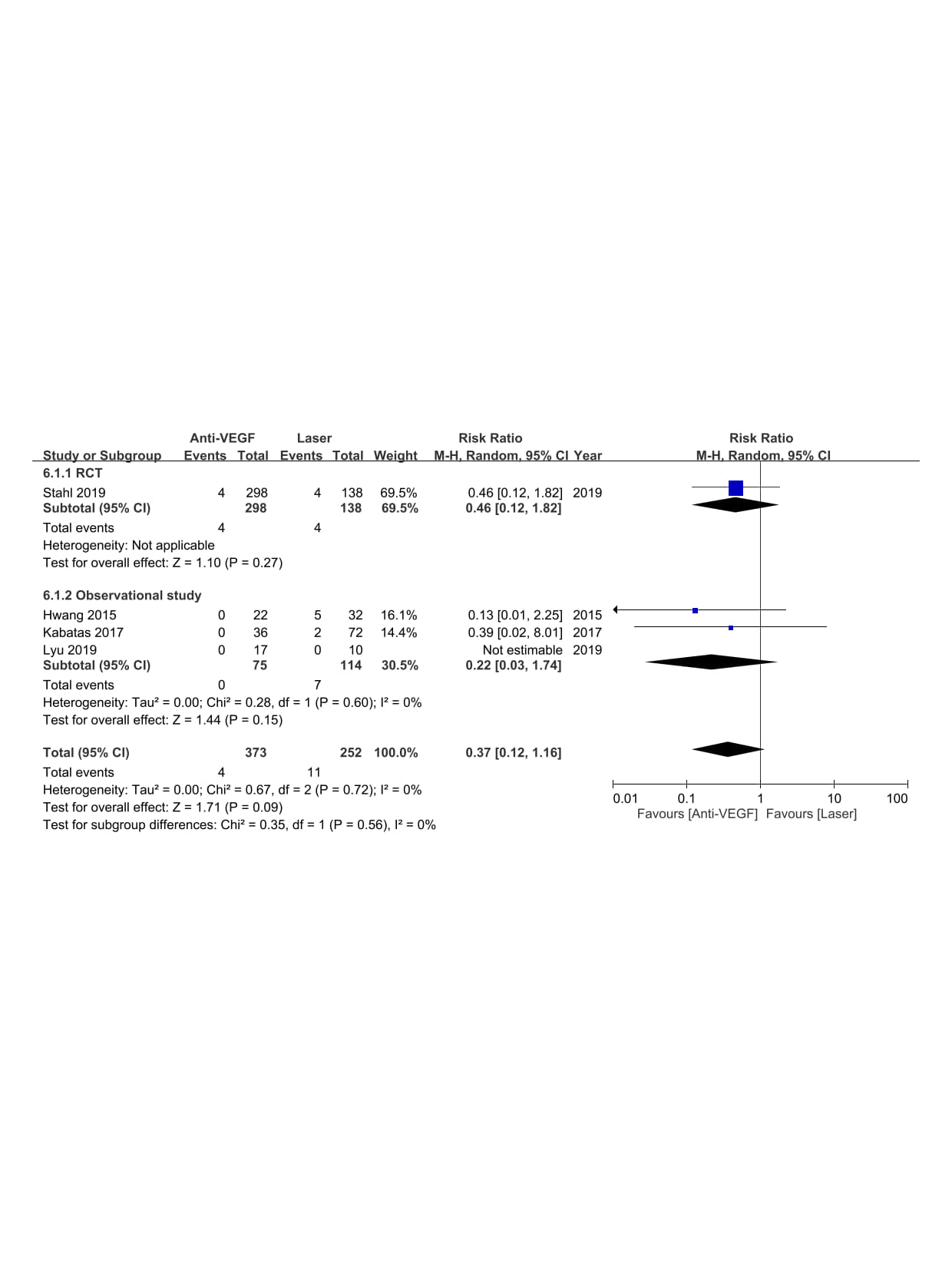


**Supplementary Figure 7.** Forest plot of the incidence of hemorrhage comparing anti-VEGF and laser groups. CI= confidence interval


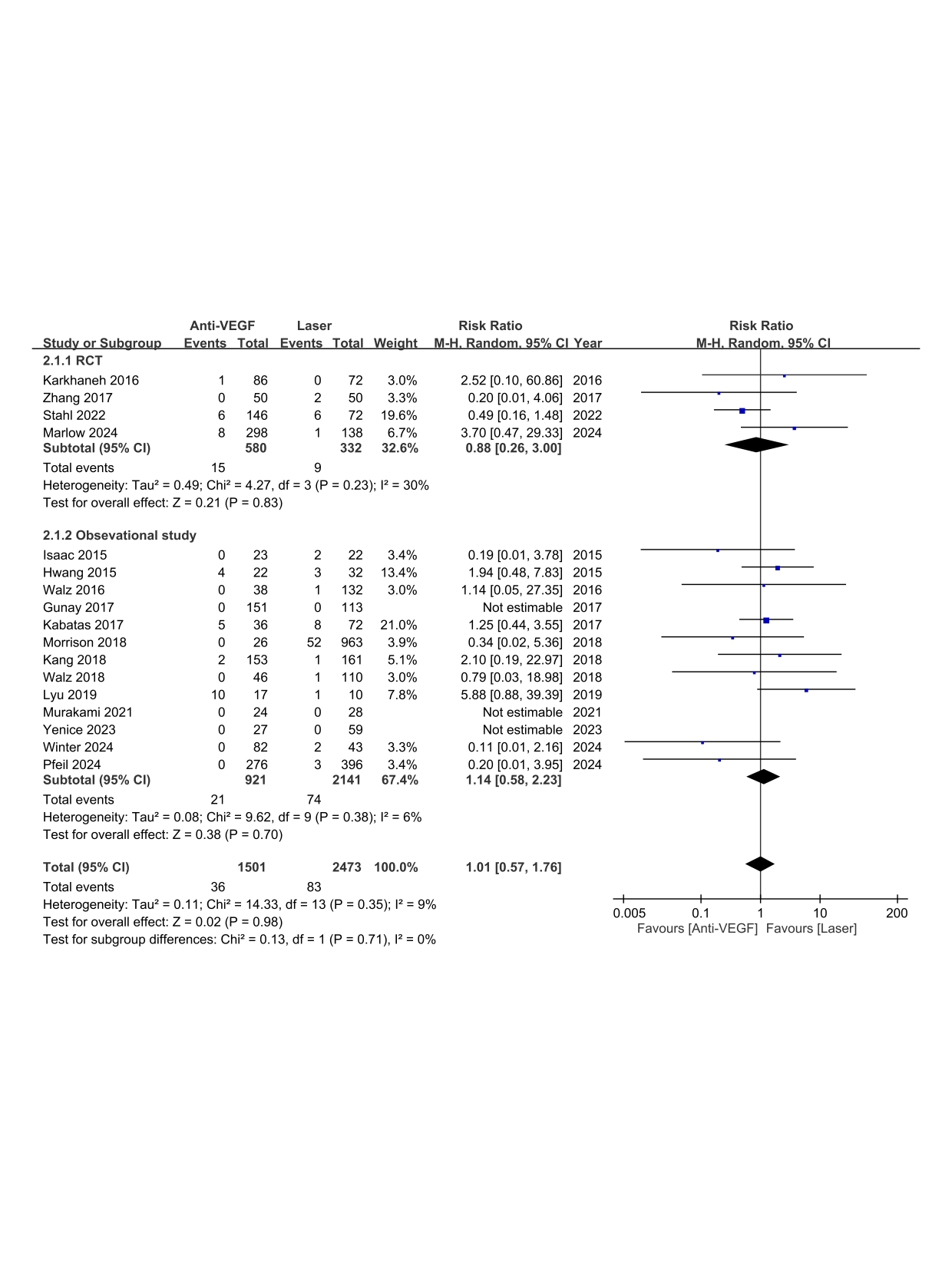


**Supplementary Figure 8.** Forest plot of the incidence of uveitis comparing anti-VEGF and laser groups.

CI= confidence interval


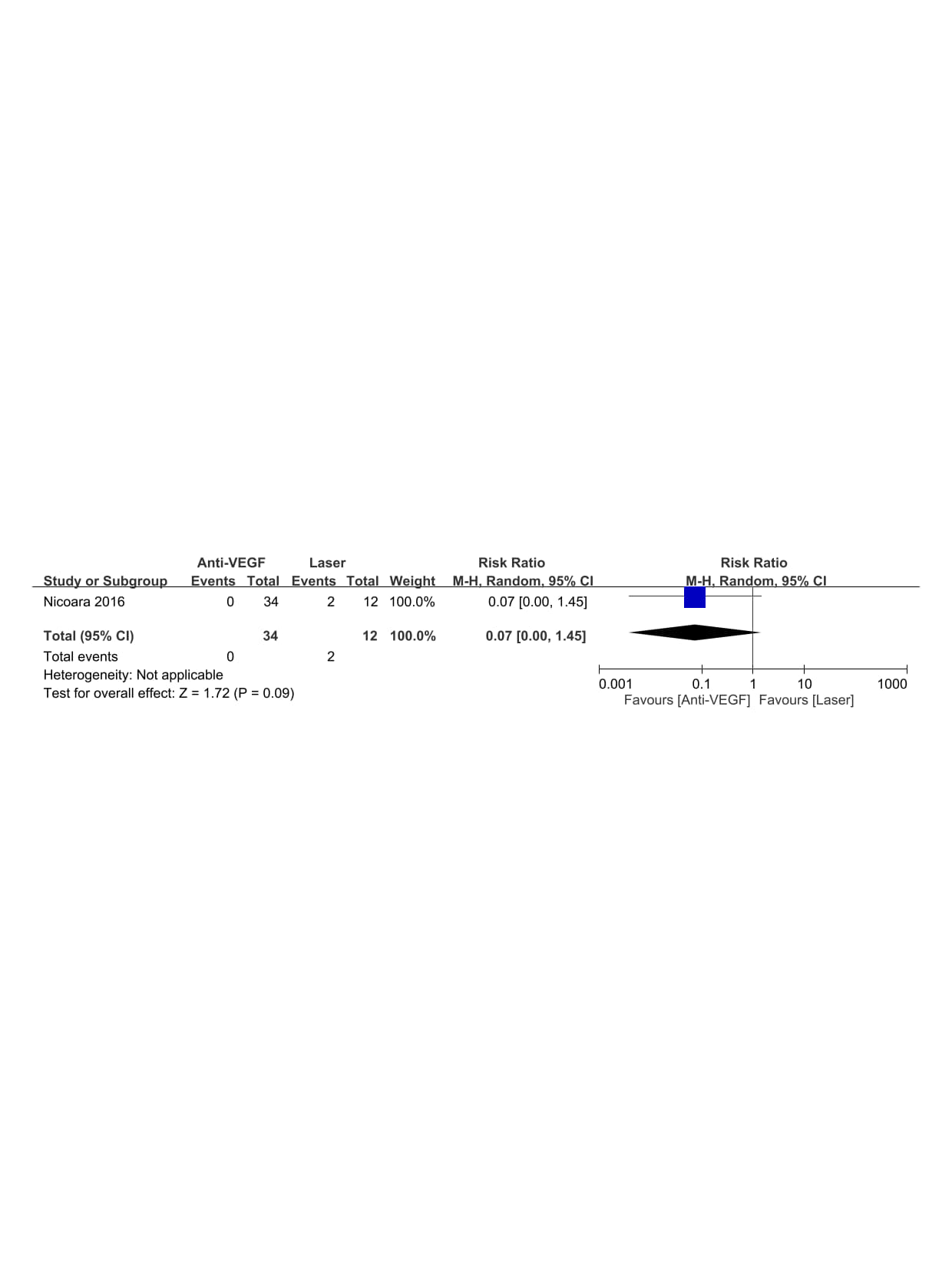


**Supplementary Figure 9.** Forest plot of the incidence of endophthalmitis comparing anti-VEGF and laser groups. CI= confidence interval


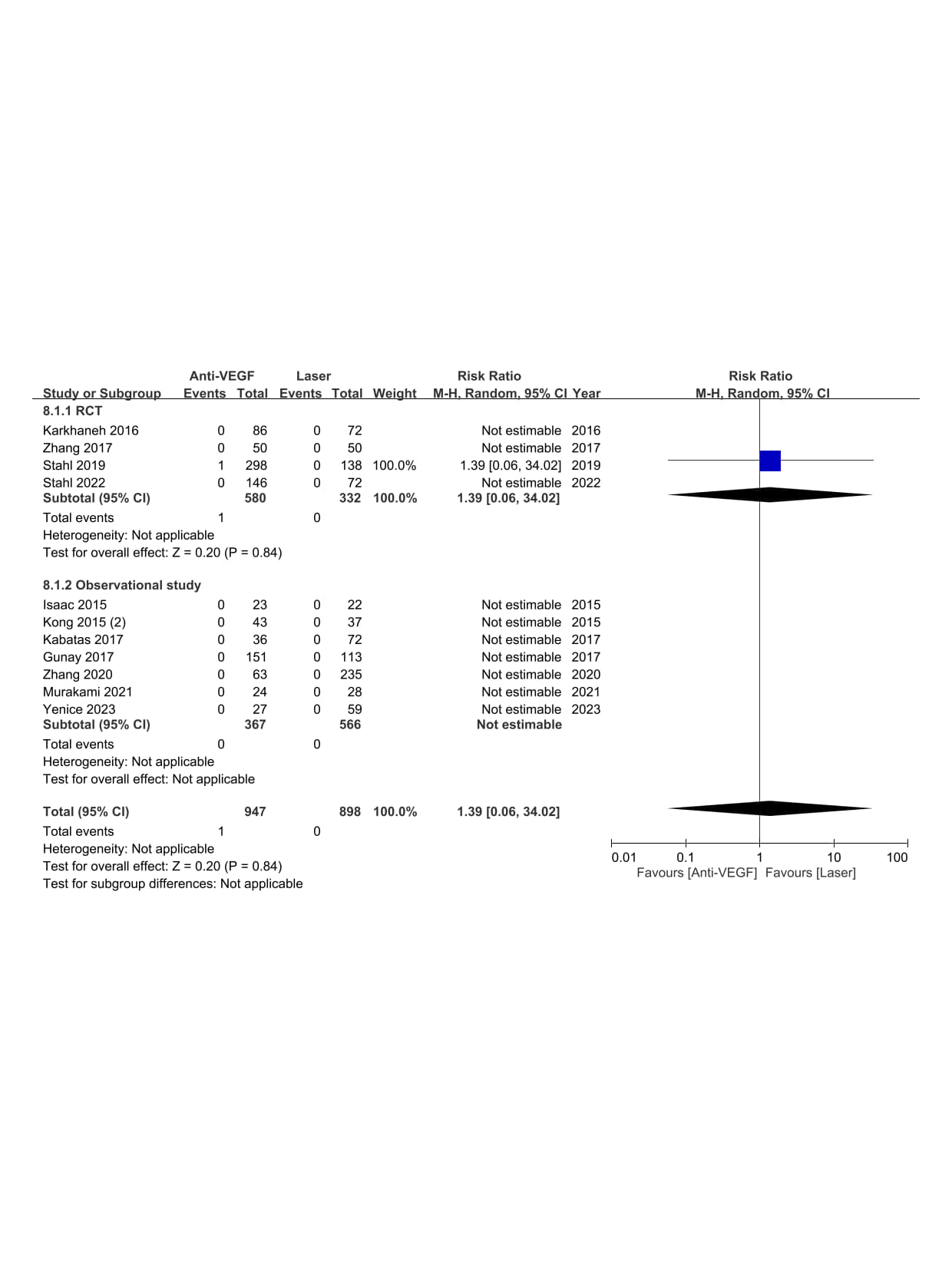


**Supplementary Figure 10.** Forest plot of the incidence of cataract or lens opacity comparing anti-VEGF and laser groups. CI= confidence interval


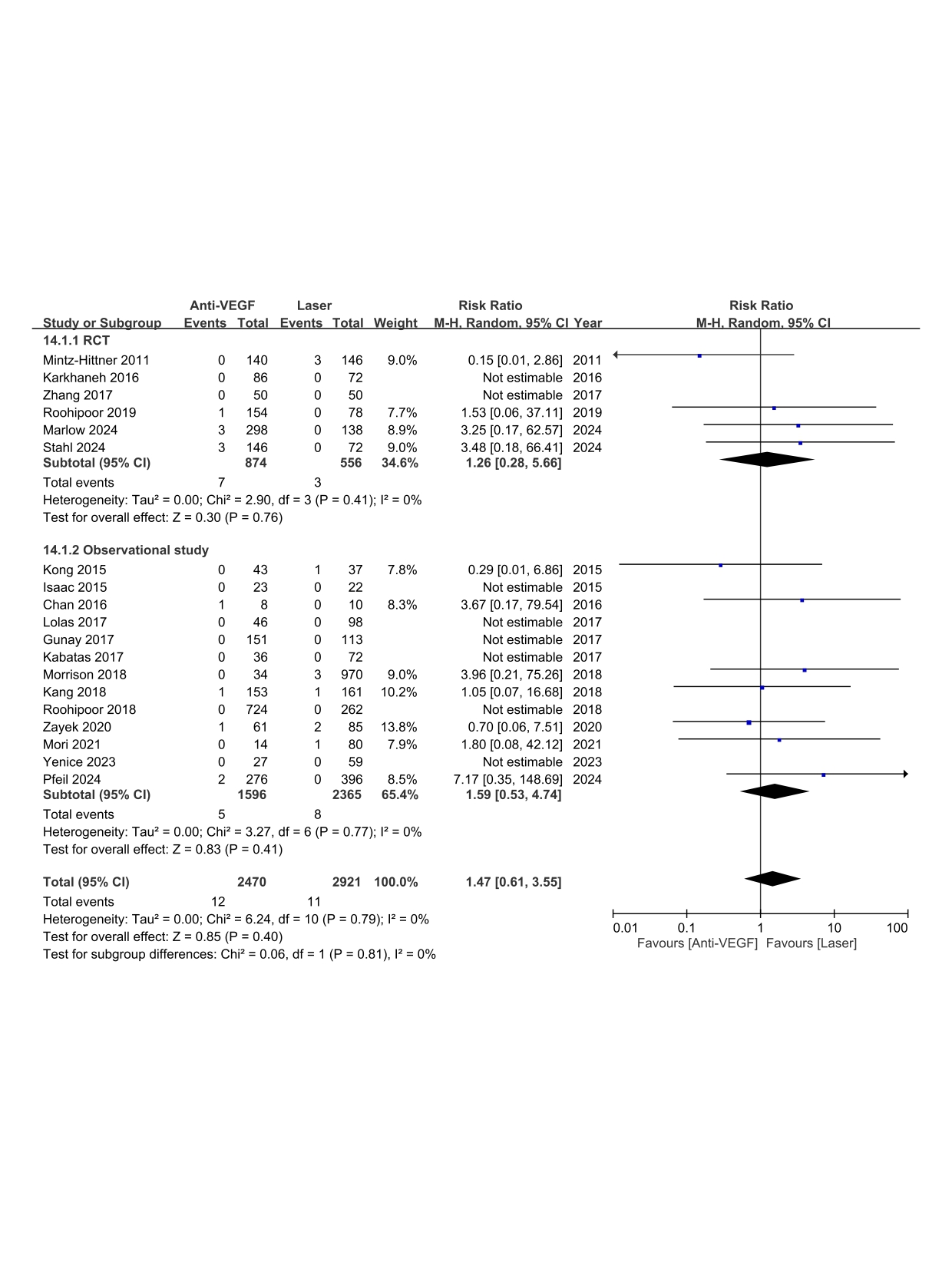


**Supplementary Figure 11.** Forest plot of the incidence of keratitis comparing anti-VEGF and laser groups.

CI= confidence interval


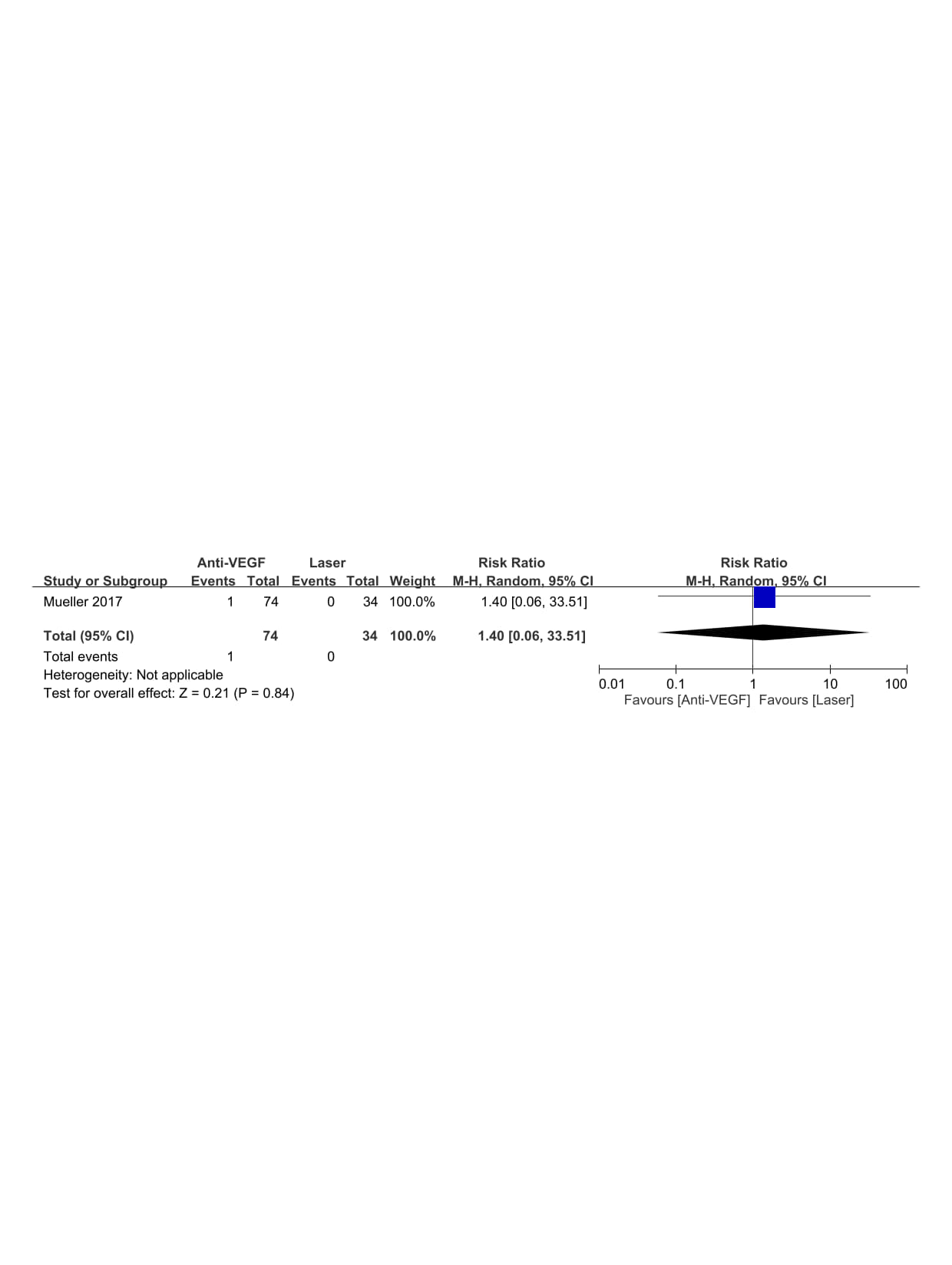


**Supplementary Figure 12.** Forest plot of the incidence of corneal erosion comparing anti-VEGF and laser groups. CI= confidence interval


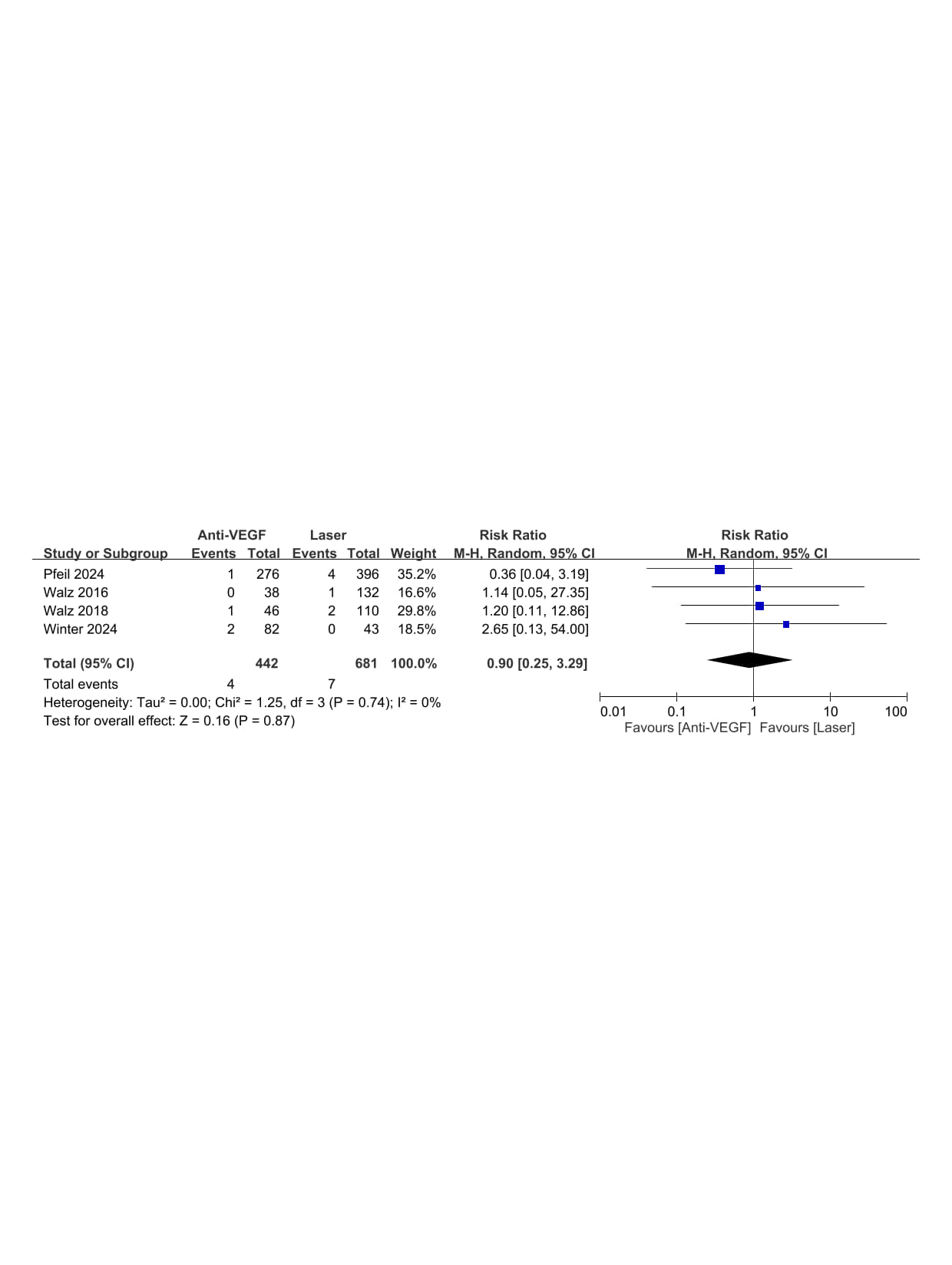


**Supplementary Figure 13.** Forest plot of the incidence of corneal opacity comparing anti-VEGF and laser groups. CI= confidence interval


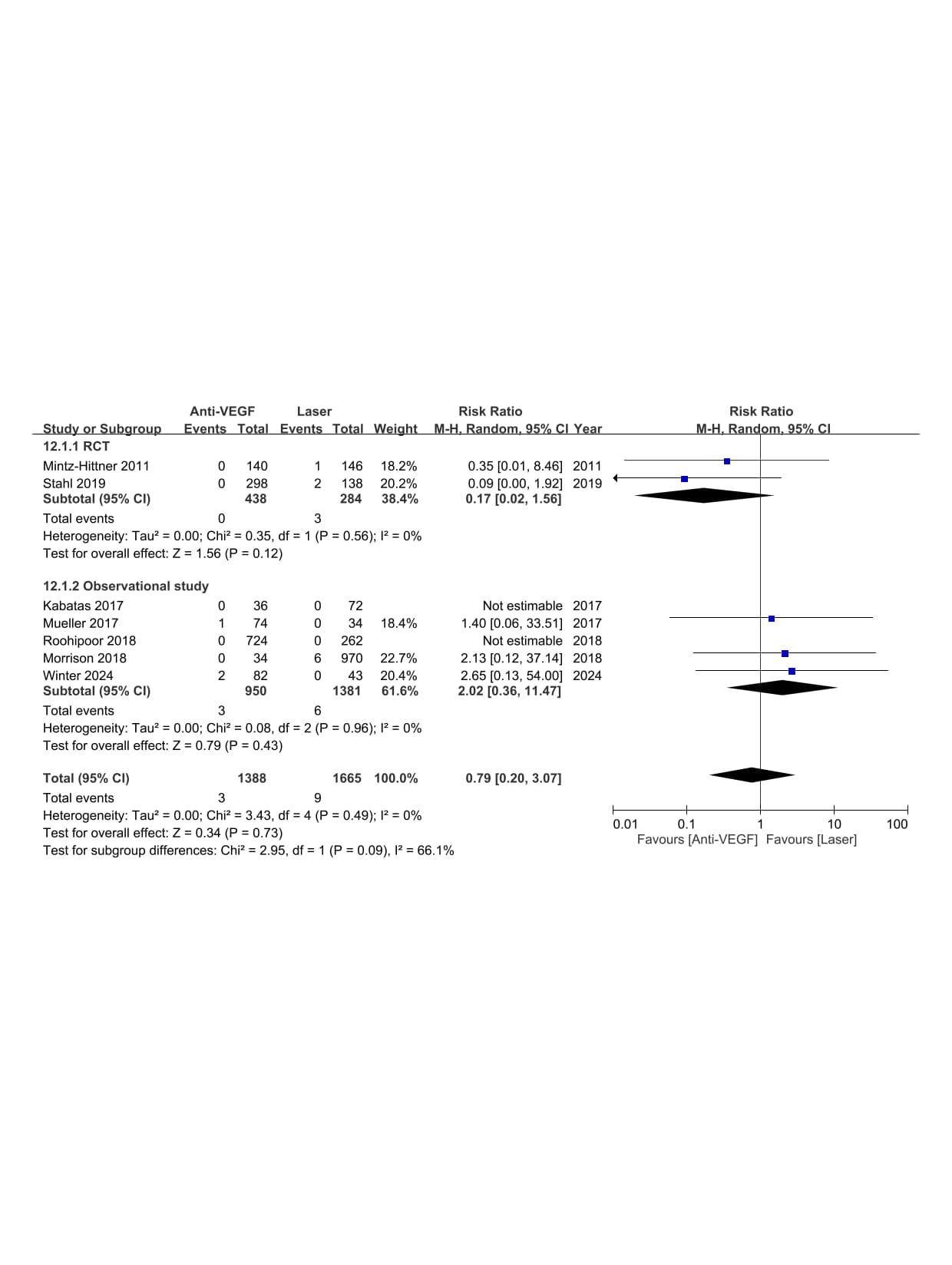

Supplement: Supplementary file 1 — Appendix S1. [file AOS-104-e1-s001.docx]
